# Calcium, Phosphate, and Vitamin D in Children and Adolescents with Chronic Diseases: A Cross-Sectional Study

**DOI:** 10.3390/nu16091349

**Published:** 2024-04-29

**Authors:** Marlene Fabiola Escobedo-Monge, Pilar Bahillo-Curieses, Joaquín Parodi-Román, María Antonieta Escobedo-Monge, Pedro Alonso-López, José Manuel Marugán-Miguelsanz

**Affiliations:** 1 Department of Pediatrics of the Faculty of Medicine, University of Valladolid, Avenida Ramón y Cajal, 7, 47005 Valladolid, Spain; 2 Section of Pediatric Endocrinology, University Clinical Hospital of Valladolid, Avenida Ramón y Cajal, 7, 47005 Valladolid, Spain; pilarbahilloc@yahoo.es; 3 Science Faculty, University of Cadiz, Paseo de Carlos III, 28, 11003 Cádiz, Spain; joaquin_parodi@yahoo.es; 4 Department of Chemistry, Science Faculty, University of Burgos, Plaza Misael Bañuelos s/n, 09001 Burgos, Spain; antoitalia777@gmail.com; 5 Section of Gastroenterology and Pediatric Nutrition, University Clinical Hospital of Valladolid, Avenida Ramón y Cajal, 7, 47005 Valladolid, Spainjmmarugan@telefonica.net (J.M.M.-M.)

**Keywords:** obesity, undernutrition, physical activity, body fat percentage, energy expenditure

## Abstract

Chronic diseases may affect the nutritional status of children and adolescents. Calcium (Ca), phosphorus (P), and vitamin D (Vit-D) are crucial nutrients for their growth and development. Proper diagnosis and treatment are critical components of personalized and precision medicine. Hence, we conducted a cross-sectional and comparative study to evaluate Ca, P, and Vit-D levels in their non-skeletal functions and their association with health and nutritional biomarkers in children and adolescents with diverse chronic conditions. We performed anthropometric, body composition, clinical evaluation, biochemical analysis, and dietary survey methods. A total of 78 patients (1–19 years, 43 females, 42 children) took part in this study. Overall, 24, 30, and 24 participants were obese, undernourished, and eutrophic, respectively. Results found that 74% and 35% of individuals had deficient Vit-D and Ca intake, respectively. Most cases were normocalcemic. Results also found that 47% of the subjects had Vit-D deficiency (VDD), 37% were insufficient, and 37% had hypophosphatemia. Of the 46% and 31% of patients with VDD and insufficient levels, 19% and 11% were hypophosphatemic, respectively. Calcium, P, and Vit-D levels were associated with anthropometric parameters, body mass index, body composition, physical activity, diet, growth hormones, and the immune, liver, and kidney systems. These results show the coincident risk of altered Ca, P, and Vit-D metabolism in children and adolescents with chronic diseases.

## 1. Introduction

Today, children and adolescents represent more than a third of the world’s population [1]. Chronic diseases, that is, chronic health conditions or long-term health conditions can affect them through multiple mechanisms [2]. There is a growing body of literature that recognizes the importance of chronic diseases in these groups due to the impact on their growth and development [1]. From pregnancy and during childhood, malnutrition can act as a risk factor for chronic diseases later in life, particularly coronary heart disease, type 2 diabetes mellitus (T2DM), and metabolic diseases. Adolescents are a nutritionally vulnerable group due to their high growth needs, dietary patterns and lifestyles, risk behaviors, and their susceptibility to environmental influences. Adolescent nutritional problems, whether malnutrition or nutrition-related chronic diseases, are mainly the result of dietary deficiencies related to physiological, socioeconomic, and psychosocial factors. Likewise, there are dietary factors also associated with increased risk of other chronic diseases such as some cancers, hypertension (HTA), and DM [3].

It is noteworthy that in 2017, more than 2.1 billion children and adolescents were affected by noncommunicable diseases (NCDs). NCDs are the result of the interaction of genetic, physiological, environmental, and behavioral factors [1]. For example, in the US (two in five students, ages 6 to 17) [4] and in the European Union (one in five students, ages 11 to 15, 19 countries) [5], children and adolescents suffer from a chronic illness such as asthma, DM, or epilepsy. Advanced technologies and novel interventions have allowed children and adolescents with NCDs to surpass these ages [6,7,8]. Nevertheless, few children experience a single NCD or risk factor, and these conditions often manifest themselves and require lifelong medical treatment [1]. According to the World Health Organization (WHO), the deleterious effects of deficiencies in adolescents and nutrition-related disorders, such as obesity, are ignored in the short/long term, possibly owing to yet insufficient data on their contribution to morbidity and mortality [3].

It is crucial to control the processes of the disease that contribute to undernutrition and overweight/obesity, risk factors for adequate growth and development. Chronic micro or macronutrient deficits can limit somatic growth and have variable direct effects on the function of the affected organ [2,9]. In this sense, calcium (Ca), inorganic phosphorus or phosphate (P), and vitamin D (Vit-D) play an essential role in growth and development and contribute to optimal peak bone mass, are at a maximum in the pediatric stage, and are significant to prevent osteoporosis into adulthood [10,11]. Phosphorus and Ca, vital for bone mineral density and non-skeletal functions, are crucial for physiological processes such as DNA structure, cell signaling, blood coagulation, muscle contraction, and neuronal excitation [12]. Furthermore, Vit-D has anti-inflammatory, insulin-sensitizing effects and regulates Ca and P balance [13]. In childhood, Vit-D deficiency (VDD) is more prevalent in chronic illnesses such as long-term systemic diseases that compromise the renal, hepatic, gastrointestinal, skin, neurological, and musculoskeletal systems [14].

Calcium and P metabolism depends on the actions of the parathyroid hormone (PTH), Vit-D, serum and urinary concentrations of Ca and P (intestinal absorption, urinary excretion, and skeleton actions) and the fibroblast growth factor (FGF23) regulated mainly in three organs: the intestine, the kidney, and the bone [15]. Guaranteeing adequate Ca, P, and Vit-D levels in young people with chronic conditions should be a priority. Nonetheless, little is known about Ca, P, and Vit-D levels in these patients. Personalized and precision medicine are helpful and require tools to improve the patient’s quality of life. We hypothesize that children and adolescents with chronic illnesses could have abnormal serum Ca, P, and Vit-D levels. Therefore, the main aim of this study was to evaluate Ca, P, and Vit-D levels in their non-skeletal functions and their association with health and nutritional indicators in a series of children and adolescents with chronic diseases.

## 2. Materials and Methods

### 2.1. Study Site, Design, and Participants

**Figure 1:**
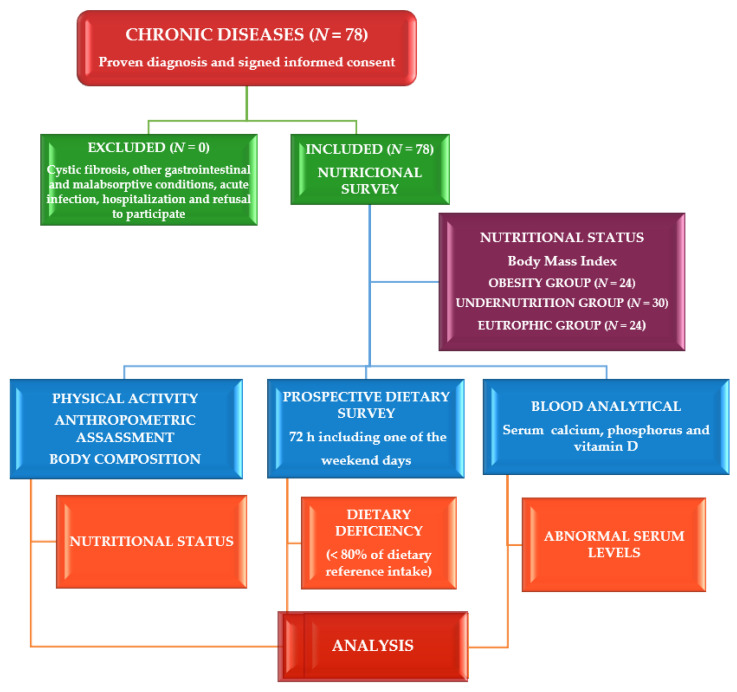
Flow diagram assignment of children with chronic disease (*n* = 78).

The design of this cross-sectional and comparative study (Figure 1) has been previously published [6,16,17]. This study was set up in the Nutrition Unit of the Pediatric Service at the University Clinical Hospital in Valladolid, Spain. The sample was obtained from the total number of cases evaluated for 18 months, successively referred to this unit. Eligible subjects were selected by systematic sampling. Patients under 19 years of age (children < 10 years and adolescents ≥ 10 years) with a history of chronic disease were the inclusion criteria. Chronic diseases include malnutrition (cause unknown), syndromic diseases, encephalopathies, renal diseases, hyperlipidemia, insulin-dependent diabetes mellitus (IDDM), and eating disorders. The exclusion criteria were patients with cystic fibrosis (CF) [18], other gastrointestinal and malabsorptive conditions, acute infection, hospitalized patients, and those who refused to participate.

### 2.2. Ethical Consideration

Ethical approval of the study protocol was obtained from the Ethics Committee of the University Clinical Hospital in Valladolid (INSALUD-Valladolid, 14 February 2002). The procedures of this study were carried out in agreement with the recommendations of the Declaration of Helsinki. Written informed consent was obtained from the relatives/guardians of all patients prior to their participation in the study.

### 2.3. Clinical Evaluation

Demographic data were collected in a questionnaire. During the clinical examination, we assessed every patient for the presence of symptoms due to altered nutrient status. Daily physical activity (PA) was assessed using a questionnaire adapted to the age of children and adolescents, based on the Global Physical Activity Questionnaire (GPAQ) [19]. We collected PA information during the week prior to each patient’s evaluation, covering occupational PA, home-based PA, and recreational PA (sports/other extracurricular activities). Total time spent on PA (TTSPA: sports, school gymnastics, and physical therapy) was recorded in hours/week. For better evaluation, patients were divided into three categories: patients very active/active, with light PA, and sedentary/very sedentary.

### 2.4. Assessment of Phenotypical Characteristics

Anthropometric measurements of weight, height, wrist, waist (WC), hip (HC), and mid upper-arm circumference (MUAC) were made using standard techniques. Using Frisancho [20] and Orbegozo tables [21], Z-score of weight for age (WA), height for age (HA), age-for-50° height (A50H), weight for height (WH), body mass index for age (BMI), BMI–height–age, nutritional index, Waterlow I and II (weight and height) [22], mid-arm muscle circumference (MAMC), fat-free mass (FFM), fat mass (FM), waist/hip ratio (WHR), and waist/height index (WHI) were calculated. Using a Holtain Skinfold Caliper (Holtain LTD, Crymych, UK), triceps, biceps, subscapular, and suprailiac skinfolds thickness and sum of skinfolds (SS) were measured. The BMI-for-age Z-score was used to categorize patients as underweight (<−2 standard deviations [SD]), eutrophic (−2 to +2 SD), or obese (>+2 SD). Body composition assessed three compartments, including FM, bone mineral content, and FFM, using anthropometric measurements and bioelectrical impedance analysis (BIA) [RJL BIA-101 (RJL System, Detroit, MI, USA)]. Body fat percentage [(BF%: fat mass/total weight) × 100] was calculated; the excess body adiposity was ≥25% for boys and ≥ 30% for girls. The fraction of body fat was estimated using the fat mass index [FMI = fat mass/height^2^ (kg/m^2^)] and the free fraction of body fat with the fat-free mass index [FFMI = fat-free mass/height^2^ (kg/m^2^)] [23]. Basal energy expenditure (EE) or at rest (REE) was measured with Indirect Calorimetry (IC) in fasting, using a canopy system under standardized conditions [Deltarac II (Datex-Ohmeda. Helsinki, Finland)].

### 2.5. Dietary Assessment

In a prospective 72 h dietary survey (including one of the weekend days), parents/guardians/patients recorded all foods eaten and their amounts (using common kitchen measurements) in the week prior to the blood test. Daily energy intake, fiber, carbohydrates, protein, lipids, monounsaturated, polyunsaturated, and saturated fats; Vit-A, Vit-B1, Vit-B2, Vit-B6, Vit-B12, Vit-C, Vit-D, Vit-E, niacin, and folic acid; and Ca, magnesium (Mg), iron (Fe), zinc (Zn), and iodine, were calculated as the percentage of Dietary Reference Intake (%DRI) or adequate intake using the Mataix Food and Health software (FUNIBER, v.1.1.5. 2005), which provided the percentage of actual nutrient intake relative to the Spanish recommendations [24,25]. The normal range of dietary intake was 80% to 120% DRI. Patients did not receive any supplementation.

### 2.6. Laboratory Exploration

Fasting venous blood samples were collected on the same day as IC and clinical evaluation. Complete blood count, biochemical profile, and acute-phase protein activity, including C-reactive protein (CRP) >4 U/L and erythrocyte sedimentation rate (ESR) in women >20 mm/h and men >15 mm/h, were measured using standardized methods. Serum prealbumin ≤18 mg/dL, albumin ≤3.5 g/dL as visceral protein reserve, transferrin ≤200 mg/dL, lymphocytes <2000 cell/mm^3^, total cholesterol (TC) >200 (mild-moderate risk) and >225 mg/dL (high risk), and low-density-lipoprotein cholesterol (LDL-C) >115 (mild–moderate risk) and >135 mg/dL (high risk), were used as cutoffs to evaluate abnormal values. Total immunoglobulin (Ig) G levels, IgG1-4, IgA, IgM, and IgE, complement C3 and C4, CD3, CD4, CD8, CD16 + 56, CD19 T-lymphocytes and CD4/CD8 ratio, insulin-like growth factor −1 (IGF-1) and insulin-like growth factor-binding protein 3 (IGFBP3), folic acid, beta-carotene, Vit-B12, Vit-C, Vit-D, Vit-E, Ca, P, Fe, and Mg [17] were measured using standardized methods. Determination of serum Zn [16] and copper (Cu) [6] was carried out in the Department of Chemistry of the University of Valladolid. The following cut-off points were used to evaluate normal/abnormal levels: for serum Vit-D, severe deficiency <5 ng/mL, deficiency <20 ng/mL, insufficiency 20–30 mg/mL, and sufficiency >30 ng/mL [26,27]. Serum Ca was corrected when the serum albumin level was <4.0 g/dL, using the following formula: Corrected Ca (mg/dL) = measured Ca (mg/dL) + [4 − albumin (g/dL) [28]; normal values ranged from 8.8 to 10.8 mg/dL [29], and hypercalcemia (plasma or serum) Ca >  10.5 mg/dL or ionized Ca >  5.25 mg/dL [30]. Serum P ranged from 4.5 to 6.5 mg/dL [18,29], hypophosphatemia < 4.5 mg/dL, and hyperphosphatemia > 6.5 mg/dL. The serum Ca/P ratio < 18 years 1.7–2.7 and >18–34 years 2.2–3.2 [31]. All were obtained by standardized methods.

### 2.7. Statistical Analysis

Participants were classified by age group according to Tanner stages in children and adolescents and by nutritional status (obesity, eutrophic, and undernutrition groups) via BMI. The primary variables were serum Ca, P, Ca/P ratio, Vit-D and dietary Ca, and Vit-D intake. Secondary variables were anthropometric and clinic evaluations, complete blood count, blood biochemistry, diet survey, body composition, and baseline EE. Results are shown as mean, median, quartiles, SD, and ranges. The duration disease (DD) is shown in months. We used non-parametric tests for variables with non-normal distribution. The two-tailed Student t-test was used to analyze unpaired or paired variables. One-way analysis of variance (ANOVA test) and Pearson’s bivariate correlation test were performed to analyze normally distributed values. We analyzed categorical results by Pearson’s Chi-square test (X^2^) with Yates’s correction and Fisher’s exact test (FET). We calculated odds ratios (OR) to estimate the magnitude of the association between exposure and disease. The effects of various factors on Ca, P, and Vit-D levels were analyzed using a linear/multilinear regression model (forward method, including only variables with a *p*-value < 0.05). While correlation explains the strength and directionality of the relationship between variables, the coefficient of determination (R^2^) shows how well the regression model explains the observed data. Data management and analysis were performed using the IBM SPSS software version 29.0 (IBM Corp., Armonk, NY, USA) to perform the statistical analysis. The significance level was established at *p* < 0.05 * and <0.01 **.

## 3. Results

The results of these patients have been previously published [6,16,17]. No patient was excluded. Table 1 presents an overview of the clinical and biochemical characteristics of the 78 patients (43 women, 55%) by BMI groups. 

The average age was 9.6 ± 4.8 (from 1 to 19 years), 54% of patients (42 cases) were children, and 46% (36 cases) were teenagers. Based on nutritional status by BMI, 24, 30, and 24 participants were obese, undernourished, and eutrophic, respectively. Overall, 30% (23 cases) of patients had polymorbidity. The obese group had higher levels of IGF-1, IGFBP3, and basal EE than the eutrophic group and both had higher levels than the undernourished group. The mean TTSPA was 3.4 ± 2.1 h/week. In total, 63% of patients were active/very active, 24% had light PA, and 13% were sedentary/very sedentary. TTSPA had an association with age (*R*^2^ = 0.157, *p* = 0.030), a positive correlation with BMI (*r* = 0.295 *, *p* = 0.019), BMI Z-score (*r* = 0.258 *, *p* = 0.041), and serum Ca/P ratio (*r* = 0.310 *, *p* = 0.015), and inverse correlation with serum P (*r* = −0.321 *, *p* = 0.012).

The diet for the entire series was hyperproteic (276% DRI) with high cholesterol intake (266% DRI), slightly low carbohydrate consumption (79.5% DRI), normal lipids (111% DRI) intake, and deficient for Zn (68%) and iodine (70%). In the whole series, mean dietary Vit-C, D, folic acid, and Ca intake were regular. Eutrophic participants had almost double the Vit-D intake than obese and underfed patients without significant differences. A total of 74% (58 cases) and 35% (27 cases) of patients had deficient dietary Vit-D and Ca intake, and 29% (23 cases) and 11% (9 cases) had high dietary Ca and Vit-D intake, respectively. The mean serum Vit-D in the whole series was in insufficient ranges. The obesity group was deficient for Vit-D compared to undernutrition and eutrophic groups with insufficient levels, without significant differences. A total of 47% (37 cases) of participants had VDD, and 37% (29 cases) were in insufficient range for Vit-D. In the whole series and by groups, the mean serum Ca, P, and serum Ca/P were normal. No patients had hypocalcemia or hyperphosphatemia. Eight patients had hypercalcemia and 37% (29 cases) of subjects had hypophosphatemia. The mean serum Ca/P ratio was 2.1, and two adolescent females (12 and 16 years) had a high serum Ca/P ratio, one was underfed (2.88), and another was obese (2.78) child.

**Figure 2:**
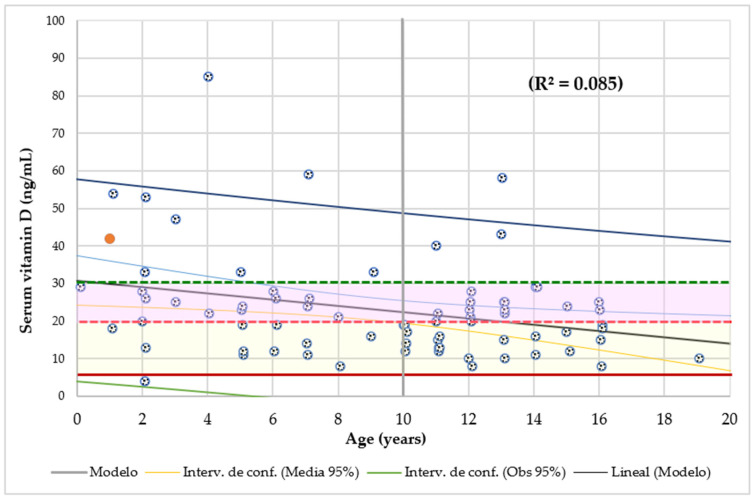
Regression of serum vitamin D by age groups. The grey line separates children from adolescents. Below the red line, the deficiency level is shown (<20 ng/mL, yellow area). Between the red and green lines, the insufficient area is shown in pink (20–30 ng/mL). Above the green line is the sufficiency level (>30 ng/mL). Below the solid red line is the severe deficiency (<5 ng/mL).

Figure 2 shows that serum Vit-D had an inverse and significant association with age. 

**Figure 3:**
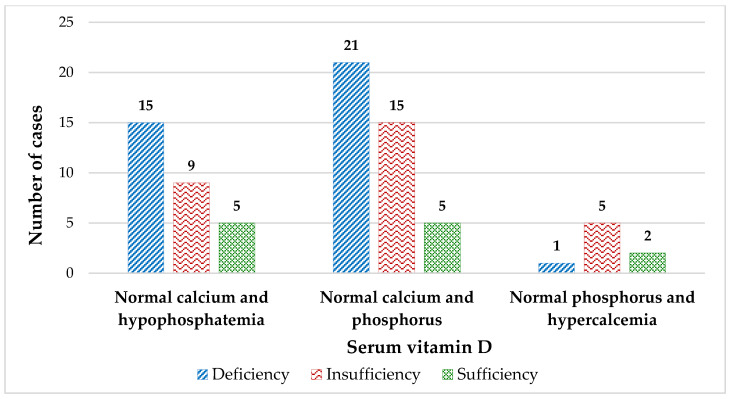
Number of cases of patients with normal serum calcium and hypophosphatemia, normal calcium and phosphorus, and patients with hypercalcemia and normal phosphatemia with respect to deficient, insufficient, and sufficient levels of vitamin D.

**Figure 4:**
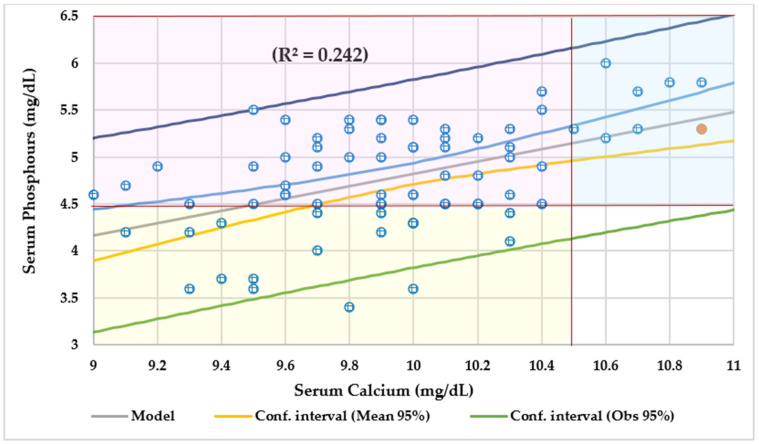
Regression of serum phosphorus by serum calcium. Red lines show normal ranges. The pink area represents normality in both nutrients, the yellow area represents hypophosphatemia, and the blue area represents hypercalcemia.

In total, 47% of patients had VDD, and 37% had insufficient Vit-D levels. One patient had severe VDD. There were no significant differences by age group. Figure 3 displays the number of cases based on serum Ca and P by serum Vit-D levels. Of the 53% of subjects with normal serum Ca and P levels, 27% (21 cases) had VDD, and 19% (15 cases) had insufficient Vit-D levels. Of the 37% (29 cases) of patients with normal serum Ca and hypophosphatemia, 19% of them (15 cases) had VDD, and 11% (9 cases) had insufficient Vit-D levels. Overall, 10% of patients (eight cases) with normal serum P levels and hypercalcemia, one patient had VDD, and 6% (five cases) had insufficient Vit-D levels. Only five patients had regular Ca, P, and Vit-D levels all at once. Figure 4 shows that serum P and Ca levels had a direct and significant association. Table 2 shows the differences among subjects with chronic conditions in the entire series. 

Based on the gender, serum Vit-D was higher in females than males. Serum and dietary Ca intake were higher in children than adolescents. BF%, FMI, FFMI, serum Ca/P ratio, IGF-1, and IGFBP3 were higher in adolescents than children. Patients with VDD had higher WA, HA, FM kg A (by anthropometry), Waterlow I, and Zn intake, and lower BF%, FMI, and serum Mg than those with normal Vit-D levels. Patients with serum P deficiency had a higher WA, HA, FFM kg A, serum Ca/P ratio and creatinine (Cr), and lower serum Ca, Cu, leucocytes, lymphocytes, Zn intake, and platelets than subjects with normal serum P. Patients with high ESR had a higher serum Ca. Patients with deficient Ca intake had a higher WA, HA, HC, BMI, FM kg, and FFM A, FM BIA, BF%, FMI, FFMI, IGF-1, serum Ca/P ratio, and lower serum Cu and P, Mg intake, leucocytes, lymphocytes, platelets, and Ca/Mg intake ratio than subjects with normal Ca intake. Table 3 displays the significant OR in the whole series.

Patients with reduced intake of lipids, kilocalories, carbohydrate, and Vit-E were more likely to have deficient Vit-D intake. Serum Vit-D deficiency was more likely in children with A50H < 5-year-old, and patients with excess body adiposity. The likelihood of finding deficient Ca intake was higher in participants with deficient Vit-A, kilocalories, Mg intake, higher weight for age, adolescents, deficient carbohydrate, and Vit-E intake, females, and children with A50H ≥ 5-year-old. The likelihood of finding hypophosphatemia was higher in patients with microcephalia, deficient intake of Vit-A, kilocalories, and Mg, and adolescents and children with A50H ≥ 5-year-old. Patients with wasting and stunting were more likely to have stunted growth and underweight, respectively.

Table S1 displays significant correlations and Table 4 the linear and multilinear regression analyses between the studied parameters and health and nutritional biomarkers. By groups, we show significant correlations (Table S2) and regression analyses (Table S3) between the studied parameters in the Supplementary Material. Serum Ca, P, and Vit-D show significant associations with anthropometric and body composition parameters, dietary intake, and biochemical and blood account biomarkers in the entire series and by BMI groups.

## 4. Discussion

To the best of our knowledge, this is the first study to explore Ca, P, and Vit-D levels and their association with health and nutritional biomarkers in a series of children and adolescents with chronic diseases. Results indicated that 47% (37 cases) of patients had VDD, and 37% (29 cases) were insufficient. Two subjects had a high serum Ca/P ratio, 10% (8 cases) had hypercalcemia, and 37% (29 cases) had hypophosphatemia. There was significant association among Ca, P and Vit-D levels and health and nutritional biomarkers studied. We need to bear in mind that the prevalence and incidence of children and adolescents with chronic conditions in EEUU are rising constantly [32], and around 40% of school-aged children and adolescents suffer from at least one chronic disease [33]. In children under 13 years of age, the prevalence ranges from 10 to 15, increasing to 30–40% in children with disabilities [34]. Calcium, P, and Vit-D, which are the nutrients’ objects of interest in this study, play important roles in the growth and development of during pediatric stage [10,11]. Even though several studies describe the prevalence of abnormal Ca, P, and Vit-D levels in specific chronic conditions, such as chronic kidney disease, conditions related to mineral and bone disorders (CKD-MBD) [35] or in CF [18] patients, little is known about these nutrients in children and adolescents with other chronic health conditions.

### 4.1. Clinical Status

Even though obesity and chronic malnutrition reflected in stunting often co-exist in low- and middle-income countries [36,37]. This dual challenge of nutritional status may happen in Western countries and in young populations with chronic conditions. Based on the WHO, the double burden of malnutrition, characterized by the coexistence of undernutrition (stunting) with overweight/obesity, can lead to diet-related NCDs in individuals, households, and populations, with repercussions throughout life [38]. In our series, fourteen individuals (18%, ten children and four adolescents) had stunted growth. Stunting after the age of six has a crucial impact on later adult height than if this situation occurs before the age of six, highlighting the importance of the growth catch-up period in subsequent development [36]. In addition, 14% of patients (11 cases) were wasting and stunting, all at once, suggesting that these patients were at higher risk of remaining malnourished.

According to the National Health Survey (2017) of the Spanish Ministry of Health, in the Spanish population from 2 to 17 years old, 13.3% were underweight, 16.2% were overweight and 10.3% were obese [39]. In contrast, in this study, the prevalence of obesity (31%) and underweight (38%) was higher. Although this situation could be because these patients suffer from chronic diseases, it is crucial to consider that the risk of malnutrition in them is substantial. Regression analysis (*p* < 0.001) shows that BMI had an association with BF% (*R*^2^ = 0.773). Both BMI (*R*^2^ = 0.995) and BF% (*R*^2^ = 0.914) had an association with FMI plus FFMI. FMI and FFMI had an association with each other (*R*^2^ = 0.744). Although in the obese group FMI had a positive and significant correlation with BF% (*r* = 0.815 **) and FFMI (*r* = 0.942 **), in the underfed (*r* = 0.988 **) and eutrophic (*r* = 0.982 **) groups, only FMI was correlated with BF%.

Interestingly, the results show that IC was inversely associated with serum Vit-D, Ca, and P. In an animal model, there was a significant association between Vit-D depletion in old rats and a decrease in whole-body EE related to a change in body composition (increase in FM and a reduction in skeletal FFM, and with compromised muscle mitochondrial activity) [40]. In 36 children (7–11 years), the authors reported that the dynamic parameters involved in Ca homeostasis influenced energy balance through multiple mechanisms involving REE. These relationships differed depending on adiposity levels and nutrient intake. A trend towards a positive association between P and REE was observed only in those with excess adiposity [41]. Postprandial EE is largely dependent on ATP production, which can be affected by P availability. In 12 healthy, lean male subjects, the REE of the protein meal was greatly affected by the P content of the meal [42]. In 60% of our patients the mean basal EE was lower than the theoretical and WHO formulas. Mean REE was significantly lower in undernourished patients in contrast with eutrophic and obese patients. The basal EE had a positive association (*p* = <0.001) with BF% (*R*^2^ = 0.603), FMI (*R*^2^ = 0.670), and FFMI (*R*^2^ = 0.720). In general, basal metabolic rate (BMR) is influenced by several factors, such as body composition (FFM and FM), as well as age, sex, and body surface area. Body fat can independently predict differences in REE between individuals because adipose tissue consumes oxygen at a rate of 0.4 mL per kilogram per minute, lower compared to lean tissue. In the BMR of obese and overweight children, FFM was found to account for approximately 60% of the variability in BMR [43].

### 4.2. Vitamin D

Several reports have shown that VDD prevention is a critical and urgent public health issue worldwide that needs to be solved [44,45,46]. Vitamin D deficiency also leads to many other diseases, most of which are related to a chronic inflammatory state [44], including impaired immunity, increased autoimmunity [47], myopathy, DM [48], osteosarcopenic obesity [49], and an increased risk of malignancies (colon, breast, skin, liver, prostate, and head and neck cancer) [50]. In childhood, it is involved in the development of serious extra skeletal health conditions, including atopy and autoimmunity [51], HTA, obesity, high-density lipoprotein, systolic blood pressure, fasting glucose, insulin resistance (IR), hyperglycemia, and dyslipidemia [52]. In children and adolescents, obesity, and chronic illnesses, such as celiac disease, CF, inflammatory bowel disease, and short bowel syndrome, as well as the use of some medications (anticonvulsants), are risk factors for developing VDD [14,53]. In its non-skeletal actions, Vit-D regulates several genes (3% of the human genome) engulfed in cellular differentiation, cell cycle control, and cellular function, exerting non-calcemic/pleiotropic effects on extra-skeletal target tissues, immune and CV systems, endocrine pancreatic cells, muscle, and adipose tissue [54], as well as in the glucose metabolism, skin, reproductive system, and neurocognitive functions [55].

Inadequate exposure to sunlight during childhood and adolescence, which can be affected by skin color, the season of the year, and the latitude of residence of the subjects or by dietary supplements [56], is a risk factor for VDD and is aggravated by a poor and unbalanced diet, in these ages, even in developed countries. Unfortunately, at these ages, the most natural sources of Vit-D (oil fish, cod liver oil, egg yolks, shitake mushrooms, and liver and organ meats) are not frequently consumed [51]. In our study, although the diet for the entire series was hyperproteic, with high cholesterol intake and normal total lipids, it was deficient in Vit-D and Ca intake. Our results agree with the 2003–2004 and 2005–2006 NHANES study on 4404 children (2 to ≤19 years), wherein authors informed that serum Vit-D was associated with the Prudent Dietary pattern (all vegetable groups, fruits, other fats, mixed dishes, fish and other shellfish, tomatoes, and meats) but not with the high-fat–low-vegetable dietary pattern [57]. While the mean Vit-D intake, in our series, was regular and 11% had high dietary Vit-D eating, 74% of patients were deficient. This result is lower than the ANIBES study (Spain, 2017), which informed that according to national (94%) and European (93%) references, the population reported intakes lower than 80% of the daily recommendations, with fish [58] and eggs (children under 10 years) [11] being the main source of Vit-D.

Curiously, no significant differences were found in the dietary intake by groups, except for higher Vit C and folic acid in the obese and eutrophic groups. Most of the patients had a normal serum Vit-C level. Serum Vit-D had a positive correlation with Vit-C in the entire series, and a significant association with serum Vit-C plus Mg, especially in the obesity and underfed groups. Also, 14% of subjects (11 cases) with lower Vit-C intake had VDD. Studies reported the critical role of Vit-D and Vit-C in the regulation and modulation of the immune system in viral infections including COVID-19 [59]. In our series, 45% of patients had deficient serum Mg [17] and 26% (20 cases) of them had both VDD and hypomagnesemia. Mg deficiency is related to Vit-D deficiency and chronic diseases, such as headache, cardiac arrhythmias, high blood pressure, sleep disturbances, depression, complications of pregnancy, IR, and abnormal glucose tolerance [60]. In people with osteoporosis, the Mg content in trabecular bone is lower. Different theories include low Mg content altering the structure of apatite crystals, reduced PTH levels, target organ resistance to PTH, and decreased Vit-D [61].

What is surprising is that although eutrophic subjects consumed almost twice as much Vit-D as obese and underweight patients and undernutrition patients had more cases with high Vit-D consumption, deficient dietary Vit-D intake was more frequent in the underfed and obese groups than eutrophic patients. Patients with reduced intake of lipids (OR 6.8), kilocalories (OR 4.5), carbohydrates, and vitamin E (OR 3.7) were more likely to have deficient Vit-D intake than those with regular intake. Vit-D intake had a positive and significant correlation with dietary Zn eating (*p* = <0.001), and VDD patients had higher Zn consumption than those with regular intake. Only the obese group had an adequate mean dietary Zn intake, with 58%, 73%, and 71% low Zn intake in the obesity, underfed, and eutrophic groups, respectively [16]. Together, Zn and Vit-D at appropriate levels maintain a healthy musculoskeletal system [62]. Zn is a cofactor that can enhance the actions of Vit-D, while Vit-D can influence Zn absorption and homeostasis by regulating its transporters [62,63]. The functions of Vit-D are regulated in part by the Zn fingers of Vit-D-dependent genes. When Vit-D binds to Vit-D receptor (VDR), it interacts with the DNA-binding domain of the Zn finger to regulate the transcriptional activation of genes to exert cellular functions [63].

Additionally, serum Vit-D had a significant association with dietary intake (Figure 2). Of the 63% (49 cases) of patients with deficient Vit-D intake, 33% of them (26 cases) had VDD and 30% had insufficient (23 cases) levels. Serum Vit-D decreases significantly with the age and VDD was more likely in children with A50H < 5-year-old (OR1.4). Similarly, in 204 children, the Vit-D levels declined gradually with age and the lowest levels were in the 10–19 years group [64]. In 21,490 Spaniard patients (La Rioja, 14–105 years, 74% women), the mean serum Vit-D was in insufficient range (18.3 ng/mL) and was significantly lower in men than in women; 30.9% of this population was VDD (<12 ng/mL) and 32.8% were insufficient (12–20 ng/mL) [65]. Although in our study the mean Vit-D level (23 ng/mL) was also in the insufficient range, the prevalence was higher; 47% (37 cases) of subjects were VDD and 37% (29 cases) were insufficient; 14% of patients had Vit-D levels <12 ng/mL and one of them had severe VDD. In contrast, in the European region, the prevalence of serum Vit-D levels < 12 and 20 ng/mL was 18% and 53%, respectively [45]. In a series of CF patients, 35% had VDD and another 35% had insufficient levels [18]. In this study, only 18% of subjects had serum Vit-D levels for adequate bone health (>30 ng/mL), which prevents bone diseases and reduces the risk of many common cancers [66]. Levels from 40 to 60 ng/mL, 10% in our study, decrease the risk of infection diseases, CVD, neurocognitive disfunction, and several types of cancer [18]. Also, in this series, serum Vit-D levels were also lower in males than in females, especially in the underfed group.

Obesity is a growing public health concern and poses a significant challenge worldwide [67]. Overweight and obesity constitute a high-risk factor for developing other NCDs such as CVD, T2DM, IR, metabolic syndrome (MetS), and even cancer at younger ages and with a greater degree of severity [6,16,17,68,69]. In our series, 24 patients were obese (67% were teenagers). Although there were no significant differences in the serum Vit-D levels by BMI groups, the mean serum Vit-D in the obesity group (17.7 ng/mL) was deficient compared to the insufficient levels in the underfed (26 ng/mL) and eutrophic (24.9 ng/mL) groups. Moreover, there were more cases with VDD in the obesity group (20%) than the underfed (14%) and eutrophic (13%) groups. The highest prevalence of Vit-D insufficiency among overweight or obese children was observed in Germany (96%), Iran (95.6%), and Canada (93%). On the contrary, in Spain, a study indicates that the prevalence of VDD in obese children was 60.4% [56]. In a meta-analysis, the relative risk in 24,600 children (0–18 years) for the association between Vit-D and obesity was 1.41 [70].

One interesting finding was that serum Vit-D levels had significant inverse associations with almost all anthropometric and body composition parameters studied (Table 4 and Tables S1–S3). Patients with VDD had higher WA, HA, FM kg A, BF%, FMI, Waterloo I, and lower serum Mg than subjects with normal levels, showing a relationship with adiposity. VDD is associated with cardiometabolic risk markers in children and adolescents, where obesity is more likely to suffer from VDD and poor metabolic outcomes [52]. In 150 obese children and adolescents, as Vit-D levels increased, anthropometric measurements were more stable and did not increase [71]. A study in 229 children (3 to 18 years) with obesity and a high prevalence of VDD revealed that lower Vit-D levels were associated with higher BMI and FM [72], as in our study. Also, in our series, subjects with excess body adiposity (OR 2.7) were more likely to have serum VDD. Adipose tissues store and metabolize Vit-D. VDR is expressed in adipose tissues and Vit-D regulates adipogenesis and the metabolic and endocrine function of these tissues, which may contribute to the high risk of metabolic diseases in Vit-D insufficiency [73]. Obesity predisposes individuals to a nonalcoholic fatty liver. The effect of sequestration and volumetric dilution, due to increased deposition of Vit-D in adipose tissue, decreases its bioavailability in obese individuals, causing a decrease in Vit-D activity in the liver [14].

Even though obesity is a global pandemic, the rate of underweight has not yet decreased enough. Childhood undernutrition remains a severe challenge and poses a medical, social, and economic burden [74]. In chronic diseases, the causes of disease-related malnutrition are multifactorial and associated with the underlying disease [75]. In our series, 39% of participants had insufficient Vit-D levels, and insufficient levels were 9% more prevalent in undernourished patients than in the obesity and eutrophic groups. In a systematic review and meta-analysis (8295 children < 12 years), participants with the lowest serum Vit-D levels showed a significantly elevated risk of wasting compared to those in the highest group [74]. In our series, Vit-D intake had an inverse and significant correlation with albumin (*p* = 0.016), and there was one patient with both hypoalbuminemia and VDD. Decreased albumin concentration is believed to affect Vit-D levels as well as its bioavailability [76]. Popovska et al. reported an important role between Vit-D, albumin, and D-dimer in the early diagnosis of the most severe COVID-19 [77]. In 73 COVID-19 adults (36 severe), the Vit-D levels predicted the worst outcome independently of their severity at presentation [78].

While in the entire series, dietary Vit-D intake only had a few associations, serum Vit-D had an inverse correlation with ferritin in the obese group; 19% (15 cases) of participants had hypoferritinemia, with 4 in the underfed group having simultaneous iron deficiency anemia (IDA). In the VDD patients, three of them had low ferritin and IDA at the same time. Hypovitaminosis D was associated with an increased (98%) risk of Fe deficiency (reduced levels of ferritin, hepcidin, and hemoglobin), and children with Fe deficiency also had a higher prevalence of VDD. In children with inflammation, higher Vit-D concentrations predicted lower ferritin concentrations than in those without inflammation [79]. Moreover, in the whole series, serum Vit-D had a significant association with Vit-B2 and Vit-B6. Riboflavin or Vit-B2, a yellow, fluorescent compound, is a precursor for the flavin mononucleotide (FMN), adenine dinucleotide (FAD), and as covalently bound flavin, participating in the metabolism of niacin, vitamin B6, folate, cobalamin, Vit-D, and choline [80].

Another finding was that Vit-D had a positive correlation with triglycerides (TG), and AST, in the entire series, and a significant association with TG in the underfed group. There were 18% and 6% of individuals with CV risk due to hypercholesterolemia and hypertriglyceridemia, in turn. Of them, eight and two subjects had VDD, in that order. In 243 children (9–18 years), patients with VDD showed higher TGs, with a higher TG/high-density lipoprotein cholesterol (HDL-C) ratio. Low levels of Vit-D could lead to increased levels of TG, TC, and LDL-C and decreased levels of HDL-C, identified as risk factors for atherosclerosis and CVD in adulthood [81]. In 2171 children from the pan-European IDEFICS/I, VDD was negatively associated with systolic and diastolic blood pressure, fasting glucose, homeostatic model assessment for IR, and TG [82]. In our series, 15% (12 cases) had high AST levels and 3 and 7 of them had deficient and insufficient Vit-D levels, respectively. In 988 adolescents at the end of Vit-D supplementation, a significant reduction in the levels of AST, ALT, direct bilirubin, total bilirubin, LDH and gamma glutamyl transferase (GGT), and an increase in total protein and albumin was observed only in the group with an abnormal value [83].

Another interesting finding is that serum Vit-D had an inverse association with IGFBP3 in the eutrophic group. In the whole series, IGF-1 had a significant association with IGFBP3 (*p* = <0.001). As it was expected, adolescents had higher IGF-1 and IGFBP3 levels than children [84]. The obese group had higher levels of IGF-1 and IGFBP3 than the eutrophic group and both had higher levels than the undernourished patients. During childhood growth and development, growth hormone (GH) induces hepatic synthesis of IGF-1, increasing the activity of P450 in hepatic mitochondria, which directly induces the 25-hydroxylation of Vit-D, increasing its serum concentration [85]. In a double-blind, placebo-controlled study with 117 children (4–8 years) at baseline, Vit-D was positively associated with muscle strength, FFMI, and IGFBP3. After 20 weeks, patients who took 20 µg/day of Vit-D3 increased their IGF-1 and IGFBP3 levels. This effect of Vit-D supplementation reinforces the hypothesis that Vit-D stimulates hepatic production of IGF-1 and IGFBP-3 and stimulates the synthesis of cytoskeletal proteins, including IGFBP-3 in muscle cells [86]. Similar results were found in a series of 22 rachitic children who received an oral megadose of Vit-D, achieving a significant increase in IGF-1, IGFBP3, and Vit-D levels [53].

Surprisingly, the current investigation found that Vit-D, in the whole series, had a positive association with leukocytes and IgG3, and an inverse correlation with IgG3 plus CD16 + 56 T-lymphocytes in the underfed group. CD16 + CD56 are a marker for natural killer (NK) cells [87]. VDR is expressed in many organs of the human body, such as in the lungs, reproductive tract, and immune system [88]. It is found in T and B cells, monocytes, macrophages, dendritic cells, and NK cells, through the enzyme 1α-hydroxylase, responsible for the activation of Vit-D precursors [87,89]. In this series, VDD patients had a significantly lower number of leucocytes, neutrophils, and IgM levels than those with sufficient Vit-D levels. In 307 infertile men, the authors reported that Vit-D had an inverse association with leucocytes, neutrophils, eosinophils, lymphocytes, and monocytes [88]. Immunomodulatory actions of active Vit-D include effects on the innate and adaptative immune systems via direct link with VDR dendritic cells and T-lymphocytes. Vit-D may reduce T helper cell differentiation and inhibit the IgM and IgG production from B-lymphocytes [89]. Moreover, Vit-D promotes survival, defending cells against harmful signals by inhibiting inflammatory responses, changing the pathways through which T-helper-2, macrophages, and regulatory T cells differentiate, maintaining energy and redox hemodynamics [50]. Vitamin D produced by monocytes or macrophages can act on activated T and B lymphocytes, regulating the synthesis of cytokines and Ig, respectively [66]. Furthermore, VDD would coincide with a shift from a Th2 to a Th1 immune response [90].

This study shows that there was an elevated prevalence of VDD (47%), especially in children and males, insufficient (37%) levels, and lower dietary Vit-D intake (74%). Vitamin D was related to adiposity, undernutrition, diet, Vit-B2, Vit-B6, Vit-C, Mg, Zn, Fe, albumin, and lipids metabolism, liver system, growth factors, and immune response.

### 4.3. Calcium

Nowadays, there is a high prevalence of schoolchildren with deficient Ca consumption, which has become a particular public health concern [91,92]. Calcium deficiency is greater in children in developing countries, affecting between 79% of children aged 10 to 16 years in Bangladesh and 100% in Senegal [93]. However, in the NHANES study from 1999–2000 to 2017–2018 (80,880 people, 2 to 60 years), worrying trends were observed between 2009–2018, with a decrease in dietary Ca intake, being of greatest concern in men, children ages 9 to 18 years, and non-Hispanic Whites [94]. Contrary to expectations, the mean dietary Ca intake in our whole series and by BMI groups was normal, without significant differences. Dietary Ca intake was inversely associated with age, especially in the underfed group. Although in our study children had higher Ca intake than adolescents, the odds of finding patients with deficient Ca intake were higher in adolescents (OR 2.9) and in patients with A50H ≥ 5 years (OR 1.4) than younger ones. Nevertheless, the results show that in the whole series, 35% of individuals had deficient dietary Ca intake (27 cases), and children under 10 years had a lower prevalence (6.5%, 5 cases) than adolescents (28%, 22 cases). In contrast, in 1176 Spanish children aged 6–9 years, from the ANIVA study, 25.8% had inadequate Ca intake with significantly higher prevalence in girls [91]. Although in the whole series and by groups under 10 years there was no significant differences by gender, males had higher diet Ca consumption than females (*p* = 0.048) in patients >10 years. The odds of having lower Ca intake were higher in females (70% of cases, OR 1.5) than males. Similarly, in 172 children (6–11 years), low Ca intake was twice as common in girls as in boys [95]. In 131 children and adolescents (10.14 ± 2.5 years), 85.5% of the subjects had Ca deficiency, with higher consumption in subjects aged 14 to 18 years [96].

In this series, the likelihood of finding deficient Ca intake was higher in patients with higher WA (OR 3.0). Dietary Ca intake had an inverse association with several anthropometric and body composition indicators, showing a relationship with adiposity. The obesity group had more cases with lower Ca intake (15%) than the eutrophic (11%) and underfed (9%) groups. While in the EsNuPI study, carried out in 1448 children (1 to <10 years), a weak positive correlation was found between Ca eating and HA Z-score [11], in the ANIVA study, Ca intake had an inverse relationship with BMI and WH ratio in girls [91]. In 355 Spanish college students (18–30 years), dietary Ca intake and muscle strength index had negative associations with FM% [97]. In contrast, Bhargava and Cao reported that children with lower Ca intake had a lower HA [98].

Another interesting finding was that patients with deficient Ca intake had higher WA, HA, HC, BMI, FM, and FFM (kg) by A, FM BIA, BF%, FMI, and FFMI than those with normal eating. In females, there was an inverse correlation between Ca intake and HA (*p* = 0.005) and WC (*p* = 0.029). Similarly, in 244 females from 12 to 19 years, an inverse correlation between Ca intake and BMI, WC, HC, and W/H ratio, was found. In a cohort of 962 school adolescents, authors suggested that low Ca intake increases adiposity among adolescents [23]. In addition, in the IDEFICS study, in 6696 children (51%f boys, 6 ± 1.8 years), authors found an inverse association between total Ca intake and BMI, WC, sum of skinfolds (SS), and FM index in boys, and only the SS and FM index in girls [99]. A meta-analysis that included 33 studies (nine in children and adolescents) found an inverse correlation between Ca intake and body weight gain in these age group, showing that Ca intake may be a benefit in obesity prevention more than treatment [97,99,100,101]. These results support the notion that overweight and obese adolescents had a lower mean Ca intake than those with regular weight [102]. Calcium may be an essential factor in the regulation of FM [103]. Dietary Ca intake could influence fat metabolism by increasing fecal excretion of fatty acids. Its anti-obesity role could be related to high intracellular Ca levels in adipocytes, especially by increasing thermogenesis, reducing lipogenesis, or inhibiting fat absorption [97,100,103]. According to Semel’s hypothesis, a higher Ca intake decreases Ca entry into adipose cells by reducing calcitriol levels [104].

Surprisingly, despite the fact that 35% of participants had deficient dietary Ca eating, no hypocalcemic cases were found. Mean serum Ca in the entire series and by groups was normal without significant differences. Serum Ca decreases significantly with age and was higher in children than adolescents [105]. Nevertheless, in several studies, Ca intake increased with age [11,106]. In this study, serum Ca did not have a significant association with dietary Ca intake. Patients with deficient intake of Vit-A (OR 6.1), kilocalories (OR 4.9), carbohydrates (OR 2.9), and Vit E (OR 2.9) had more probability of having deficient Ca intake. In the entire series, 28% (22 cases) of patients had simultaneously deficient Vit-D and Ca intake, and 19% of patients (15 cases) with deficient dietary Ca intake had deficient serum P levels. Simultaneously, 13% (10 cases) and 15% (12 cases) of patients with lower dietary Ca and Vit-D intake had VDD and insufficient/sufficient Vit-D levels, respectively. Contrary of our results, in 172 Ukrainian children, 33% of them had insufficient Ca and/or Vit-D intake, 27% had low whole blood Ca levels and 48% had low Vit-D levels [95]. PTH mobilizes Ca indirectly by stimulating an alpha-hydroxylase that increases the production of Vit-D3, leading to increased Ca absorption in the intestine [107].

Another interesting finding is that in our series, 15% of participants had high dietary Ca consumption and eight subjects had high Ca levels (≥10.5 mg/dL) [30]. Nevertheless, this prevalence would change depending on the range used, for example, if we used the cut-off point of 10.2 mg/dL for children ≥ 12 years of age [105], we would only have three patients with hypercalcemia. In this study, there were no patients with hypercalcemic symptoms of hypotonia, poor nutrition, vomiting, constipation, abdominal pain, lethargy, growth retardation, polyuria, dehydration, and seizures [108]. Hypercalcemia is a more infrequent finding in children than adults [105] and no patients had serum Ca levels > 11 mg/dL [109], the highest cut-off point for many authors to identify hypercalcemic children [30]. Moreover, although Vit-D intoxication is another cause of hypercalcemia in childhood [109], in our study, only two out of eleven patients with Vit-D levels in the range of 30–85 ng/mL had hypercalcemia (Figure 3). In general, vitamin D-associated hypercalcemia occurs only when large doses of vitamin D are ingested [110] and is rare, as in sarcoidosis or other granulomatosis [111].

As far as body composition is concerned, serum Ca showed engaging relationships. In the whole series and by groups, serum Ca had an inverse association with almost all anthropometric and body composition parameters studied. In males, serum Ca had an inverse correlation with HA Z-score (*p* = 0.016), and in females, an inverse correlation with HA (*p* = <0.001), BMI (*p* = 0.024), wrist perimeter (*p* = 0.028), WC (*p* = 0.003), and HC (*p* = <0.001). Several studies suggest that serum total Ca may be a non-traditional risk factor for EBP along with high BMI in childhood [112,113]. In adults, several studies have shown that higher serum total Ca concentrations and albumin-corrected Ca were associated with a higher prevalence of DM because serum Ca is associated with IR in adipocytes and skeletal muscle due to a reduction in glucose transporters [114].

This study found that 26% of patients had inadequate Mg intake, 45% had hypomagnesemia, and 12% hypermagnesemia. In our series, 54% and 90% had an elevated serum Ca/Mg ratio and a low Ca/Mg intake ratio, respectively [17]. On the contrary, the ANIBES study reported that the mean Mg (79%) intake in citizens (9–75 years) was low (<80%) and was below the national RDA [58]. Even though studies had reported that Ca and Mg intake increased with age [11], this was not found in our series [17]. In the entire series, patients with deficient Ca intake had a lower intake of Mg and Ca/Mg intake ratio. Patients with deficient eating of Mg (OR 4.2) had more probability of having a deficient Ca intake. There was a positive association between dietary and serum Ca with Mg and their respective Ca/Mg ratios. Magnesium, a physiological antagonist of Ca channels, affects processes regulated by intracellular Ca concentration fluxes, being crucial for normal neurological and muscular function [115]. Poor Mg intake can increase the risk of NCDs [17,116]. Likewise, in serum, low Mg levels or high Ca levels may contribute to CVD risk, MetS, and DM [117]. Magnesium regulates Ca-Mg exchanges through the N-methyl-D-aspartate (NMDA) receptor, stabilizing the nerve fiber membrane and reducing its excitability. Decreased Mg levels increase intracellular Ca through NMDA, resulting in hyperexcitability and hypermuscular contractility, leading to HTA [118].

In this series, patients with deficient Ca intake had lower serum Cu and P concentrations. A total of 22% of the subjects had abnormal mean serum Cu, 13 cases had hypercupremia, and 4 cases had hypocupremia. In 180 preschool children (2–5 years), there was a negative association between serum Ca and Cu only in male children [119]. In this study, only two subjects had normocalcemic, hypocupremia, and low Ca intake all at once [17]. Copper is a cofactor of various enzymes (cytochrome C oxidase, superoxide dismutase, dopamine β-hydroxylase, tyrosinase, and ceruloplasmin) that participate in the processes of oxidative phosphorylation, antioxidant capacity, neurotransmission, amino acid metabolism, and conversion of ferrous Fe into ferric Fe. Calcium and Cu share the function of bone tissue formation and maintenance, with Cu being a cofactor of lysyl oxidase, an essential enzyme for bone collagen formation [119].

This study also found that serum Ca had a positive association with total protein and albumin, and a negative relationship with ferritin, glucose, Cr, and ALT. Calcium metabolism is linked to glucose metabolism by an appreciation of the biological effects of the osteoblast product, osteocalcin. Two major metabolic pathways seem to be linked to the Ca/bone and glucose/insulin [120]. Ca-dependent enzymes and Ca channels are responsible for glucose uptake after insulin stimulates muscle cells. Calcium disturbance leads to the impaired insulin secretion of β-cells [114]. An inverse association was found between Ca intake and ferritin, in 1080 girls and 524 women, and transferrin saturation as a measure of short-term Fe level was inversely associated with Ca intake [121]. In addition, transferrin had a meaningful association with serum Ca in the underfed group. A series of CF patients also showed this association [122]. Transferrin is a glycoprotein that plays a central role in Fe metabolism and is responsible for the supply of ferric ions [123]. Calcium signaling through the proteins CAMKK2 and CAMK4 (calcium/calmodulin-dependent protein kinase kinase) affects Fe transport mediated by the protein transferrin [124]. In an animal model, a Ca supplement improved the anthropometric parameters and reduced the levels of cholesterol, TG, free fatty-acid, and hepatic enzymes (ALT, AST, and alkaline phosphatase) [125].

Surprisingly, in the entire series, dietary Ca intake had an inverse correlation with IGF-1, and regression analysis shows that Ca intake was associated with IGF-1. Participants with deficient dietary Ca intake had higher IGF-1 than patients with regular intake. In NHANES III, in 5368 adults > 20 years, the results showed that Ca had a positive and significant association with IGF-1 and IGFBP3, especially women [126]. In 747 Chinese children and adolescents with short stature (<−2 SD), the serum Ca was associated with IGF-1 levels [127]. The activity of IGF-1 is regulated by IGFBPs. IGFBP-3 forms a ternary complex with IGF-1, extending its half-life in the circulating system. Therefore, IGFBP-3 acts as a stabilizer and transporter of IGF-1 [128]. Protein and micronutrients such as Zn, potassium, Mg, and Vit-D affect GHs and IGF-1 [129]. PTH induces IGF-1 synthesis to stimulate osteoblast proliferation and differentiation, indirectly stimulating osteoclast activity. GH stimulates osteoblast proliferation and collagen production by increasing the production of IGF-1 and IGF-binding protein [130].

The results show that patients with high ESR had higher levels of serum Ca. Calcium signaling is essential for the immune response. In lymphocytes, regulated increases in cytosolic and organelle Ca levels control metabolism, proliferation, differentiation, the secretion of antibodies and cytokines, and cytotoxicity [131]. In this series, although dietary Ca intake had a positive association with leucocytes and lymphocytes, and a negative relationship with CD16 + 56 T-lymphocytes, patients with deficient Ca intake had lower amounts of leucocytes, lymphocytes, and platelets. In lymphocytes, dynamic Ca changes regulate cellular functions on different time scales. Within seconds or minutes, the increase in Ca after antigen receptor stimulation affects the release of cytotoxic granules by CD8+ T cells and NK cells or the migration of lymphocytes. Hours later, Ca signals promote de novo gene expression and the production of cytokines, chemokines, cell surface receptors, or pro- and anti-apoptotic genes that shape lymphocyte function. Days after stimulation, Ca signals modulate the expression of genes that determine the differentiation of T and B lymphocytes [132].

These findings broadly support that there was a high prevalence of deficit Ca intake (35%), especially in female adolescents, and few cases with hypercalcemia. Calcium was related to adiposity, Mg, Cu, and Vit-D metabolism, total protein, albumin, transferrin, glucose metabolism, growth factors, liver and renal systems, and immune response.

### 4.4. Phosphorus

Interestingly, in our study, the mean serum P levels (4.8 mg/dL) were normal and there were no significant differences by BMI groups. Age had an inverse correlation and significant association with serum P in the entire series and in all groups. These results support evidence from previous observations that phosphatemia decreased with age [18,133,134,135,136]. Serum P levels vary depending on several factors such as bone mineralization, growth, age [134], and chronic conditions. In young patients, it is crucial to consider P levels based on age since as long as it has a lower regular value at birth, it is nevertheless higher than the upper regular level in adulthood [133]. These higher serum P levels in newborns than in older children and adults are due to a bigger fractional P reabsorption due to an even higher glomerular filtration rate. In mid-childhood, the serum P values decrease to adult values by late adolescence [137]. In this study, no gender differences in the P levels were found. This result agrees with other authors [138,139]. Nevertheless, in 15,513 individuals of the Third NHANES study, the mean serum P levels were significantly higher in women than men [140].

Surprisingly, even though two eutrophic children were CKD males, no patients had hyperphosphatemia in our study. Hyperphosphatemia is commonly seen only in the later stages of CKD. FGF23 and PTH increase the fractional excretion of P per functional nephron, compensating for the progressive loss of functional nephron mass. As CKD progresses, these mechanisms fail to overcome dietary P intake, resulting in high serum P levels [141]. Instead, 37% (29 cases) of individuals had hypophosphatemia. One of them was a very active 10-year-old CKD boy with both hypophosphatemia and VDD. Another sedentary 9-year-old CKD boy had normal Vit-D and P levels. Both followed their CKD treatment. In the same way as serum Ca, depending on the cut-off used, the prevalence of hypophosphatemic patients changes. There is no universally accepted definition of chronic hypophosphatemia. In a retrospective–prospective study on 29,279 serum P tests from 21,398 Spanish children (1 to 16 years), 1.2% of them (268 cases) had at least one result with hypophosphatemia (serum P < 4 mg/dL in children from 1–11 years and <3 mg/dL in children > 11 years [134]. Likewise, out of 349 critically ill children admitted to the PICU, 71.6% had hypophosphatemia (serum P < 3.8 mg/dL in children < 2 years and <3.5 in children ≥ 2 years). In this study, only one patient had hypophosphatemia using these ranges [142]. This fact might point out the importance of knowing the respective regular and risk levels in children and young people. The KDOQU guidelines recommend for CKD children and adolescents that P levels should be lowered toward the normal range [137].

Even though serum P levels were significantly higher in children (mean 4.9 mg/dL) than teenagers (mean 4.6 mg/dL), adolescents (OR 1.8), and children with A50H ≥ 5-year-old (OR 1.4) had more likelihood of having lower serum P levels. In our own previous series of CF patients, 18% of them had hypophosphatemia [18] probably caused by intestinal malabsorption, internal distribution, and augmented urine P losses [143]. In our current series, patients with a deficiency of Vit-A (OR 3.6), kilocalories (OR 3.4), and Mg (OR 3.4) intakes had more odds of having hypophosphatemia. Two observational studies reported that a higher proportion of plant to animal protein provided a lower P intake [144]. In addition, in this study, serum P had a positive correlation with Ca intake, especially in the underfed group and a significant association with serum Ca (Figure 4). Dietary Ca intake was positively associated with P levels in children [139]. In contrast, Jafari Giv et al. did not find any relationship between Ca and P intake and serum Ca and P levels [145].

The literature reports an inverse association between serum P and BMI, WHR, WC, and FM in specific populations such as in subjects with nonmorbidly obesity, HTA, and MetS [146,147,148]. In this series, serum P had an inverse association with several anthropometric and almost all body composition parameters studied, especially in the obese group. However, the eutrophic group had more patients with hypophosphatemia than other groups. Regulation of food intake, thermogenesis, PA capacity, and EE mediate the negative relationship between P level and weight gain [149]. Hypophosphatemic patients had higher WA, HA, and FFM kg A than subjects with normal serum P. Similarly, in 1676 postmenopausal women and 323 community-dwelling men without active disease informed an inverse association between serum P and BMI and FM [150]. In 9202 adults (45–100 years), authors found an inverse association between serum P and BMI and total adiposity reflected by fat% in both gender but stronger in women [146]. In contrast, another study suggested that serum P was associated with FM distribution rather than with obesity by itself. WH was higher than BMI as a determinant of serum P [147]. In 46,798 South Korean adults (>20 years) without previous comorbidity, serum P had a negative correlation with WC and BMI. After adjustment for age, sex, and Ca levels, the association with WC remained strong, but with BMI, it did not remain significant [136].

In the entire series, serum P had a meaningful inverse association with IGF-I, especially in the obese group. Phosphate homeostasis is regulated by a complex network of factors. The phosphatonins secreted frizzled related protein 4, and IGF-1 modulate P levels [139]. In the X-linked hypophosphatemia population, the increase in serum P level after GH treatment may be related to IGF-1 [151]. In 747 children with HA < −2 SD, serum P had a non-linear relationship with IGF-1 and the IGF-1 increase with the increase in serum P when the serum P level was >3.9 mg/dL [127]. Endocrinopathies, tumors, and VDD were the most common medical conditions associated with hypophosphatemia, finding a correlation between glycemia and phosphatemia in DM patients [134]. In the eutrophic group, serum P and the Ca/P ratio had a significant association with glucose, and in the whole series, serum P had an association with AST and Cr. Adequate P content ensures a balanced lipid profile by regulating fatty acid biosynthesis, oxidation, and bile acid excretion [149]. Hypophosphatemia and hypercalcemia may occur at the same time [114].

Interestingly, in the hypophosphatemic patients, 29% (23 cases) and another 18% (14 cases) had deficient dietary Vit-D and Ca intake, respectively. Simultaneously, there was hypophosphatemia in 19% (15 cases), 11% (9 cases), and 6% (5 cases) of individuals with VDD, insufficient Vit-D levels, and sufficient Vit-D levels. Vitamin D deficiency causes hypocalcemia and hypophosphatemia. Hypocalcemia stimulates the release of PTH, which partially corrects hypocalcemia but increases urinary P excretion, leading to hypophosphatemia [152]. Since Vit-D stimulates P absorption by decreasing PTH levels and Vit-D deficiency leads to decreased intestinal P absorption, this results in hyperparathyroidism (HPT) and increased renal P excretion mediated by PTH [153]. In addition, in our study, serum P and the serum Ca/P ratio had an inverse correlation with Cr.

Another interesting result is that serum P had an inverse correlation with CD16 + 56 T-lymphocytes and a positive correlation with leucocytes, lymphocytes, and platelets, and with monocytes in the eutrophic group. Hypophosphatemic patients had a lower number of leucocytes, lymphocytes, and platelets. In adults, hypophosphatemia may impair the chemotaxis, phagocytosis, and bactericidal activity of macrophages, causing ATP depletion, organ dysfunction, and, especially, muscle weakness [154]. In animal models, P plays a crucial role in improving the immune system (promoting a healthy microbial environment in the gastrointestinal tract), acting as a buffer for potential pathogens, and showing positive effects against pathogenic microorganisms [155]. Phosphorus deficiency can lead to a decreased number of the peripheral blood T-lymphocyte populations, affecting the cellular immune system in layers, and impairs the phagocytic ability of polymorphonuclear leucocytes [156].

These results corroborate that there was a high prevalence of hypophosphatemia (37%) especially in adolescents. Serum P had an association with diet, adiposity, growth factors, Ca, Vit-D, and glucose and lipid metabolism, liver, kidney, and immune system.

### 4.5. Serum Calcium/Phosphorus Ratio

Regarding the homeostasis of Ca and P, these elements are closely interconnected since they act among themselves by modulating various hormones, so their serum levels are approximately inversely related [157,158]. While authors suggest that adequate Ca and P intake should be in the proper ratio of 1:2 [159] to reduce the risk of poor Ca absorption [11,91], little is known on the serum Ca/P ratio. In our study, the mean serum Ca/P ratio of 2.1 was normal and there were no significant differences by BMI groups and gender. Serum Ca/P ratio had a positive correlation and association with age in the entire series and in all groups and was higher in adolescents than children. Similar results were found in a series of CF patients [18]. In adults, this ratio has been used to diagnose primary hyperparathyroidism (PHPT), especially in the subgroup of normocalcemic PHPT patients, its value being higher compared to serum Ca/corrected Ca. The Ca/P ratio over 3.5 mg/dL is an accurate, highly sensitive, and specific indicator for PHPT diagnosis [158], with 90.5% sensitive and 93.2% specific (3.35 mg/dL) [160]. In addition, a serum Ca/P ratio lower than 2.32 mg/dL can be used to diagnose patients with hypoparathyroidism [157,161]. Considering these ratios, in our series, no patients had a serum Ca/P ratio less than 2.3 mg/dL or more than 3.5 mg/dL, but two individuals had a high serum Ca/P ratio based on the cut-off points used in this study [31].

Additionally, the serum Ca/P ratio had a positive association with TTSPA, especially in the obese group. Calcium–phosphate homeostasis, strictly regulated within a very narrow range, is fundamental in the functioning of all cell types, both striated skeletal muscle and cardiac muscle, as well as in neuronal and neuromuscular activity. This homeostasis is essential for PA and exercise performance [162]. In 10 healthy and physically active Caucasians (26 ± 5 years), exercise did not affect albumin-adjusted Ca levels when comparing an exercise group to a control [163]. The mean TTSPA was 3.4 ± 2.1 h/week. This total time had a positive and significant correlation with BMI (*p* = 0.019), BMI Z-score (*p* = 0.041), and serum Ca/P ratio (*p* = 0.015), and inverse association with serum P (*p* = 0.012). Log regression between age and TTSPA show positive and significant association (*p* = 0.028) and with serum P. A sedentary lifestyle and little outdoor PA, representing 13% of participants in our series, are known risk factors for obesity, predisposing to reduced exposure to sunlight and low cutaneous synthesis of Vit-D [14]. In our series, boys < 10 years were more very active than girls but adolescent females were more active than their peers.

Interestingly, the serum Ca/P ratio had a meaningful inverse association with serum P in the entire series and by groups. Furthermore, in contrast to associations observed in serum P, the serum Ca/P ratio had a positive correlation with WA, HA, A50H, head perimeter, WC, HC, triceps skinfold Z, score, FM and FMF kg A, FM and FFM BI, arm area, MAMC (significant association), MA/FA index, and MFC in the whole series. Furthermore, there was an association between serum Ca/P ratio and HC, FM, MAMC in the obese group, with BF% in the underfed group. With triceps skinfold Z-score, FFMI, and arm fat area in the eutrophic group, and with serum P in all groups, and with protein intake in obese and underfed groups. Similarly, in a series of CF patients, serum Ca/P ratio had a positive correlation with WA, HA, HC, FFM kg A and FM BIA [18]. In addition, serum Ca/P ratio had a significant association with AST, lymphocytes, DD, protein, and Zn intake. There was an association between serum Ca/P ratio with leucocytes and Cr in the underfed group, and with kilocalories and glucose in the eutrophic group, and with protein intake in the obese and underfed groups.

At this point, it is crucial to consider several aspects to highlight. First and foremost, in this series, 15% of children and adolescents with chronic diseases suffer from a double burden of malnutrition; most of them (84%) had an increased risk of persistent hypovitaminosis D (47%), especially in children and males, and insufficient vitamin D levels (37%). This risk may be due to the high prevalence of lower dietary intake (74%), showing an alteration in Vit-D metabolism. Secondly, there was an imbalance in the Ca and P metabolism, caused on the one hand by a lower dietary Ca (35%) intake, particularly in female adolescents, and on the other hand by hypophosphatemia (37%), principally in adolescents. Thirdly, the serum Ca/P ratio was regular and had an inverse relationship with serum P. Finally, both Vit-D and Ca and P levels were related to adiposity with the risk of imbalance in body composition. Calcium, P, Ca/P ratio and Vit-D had significant associations with anthropometric and body composition parameters, especially with adiposity, dietary intake, Vit-B2, Vit-B6, Vit-C, Fe, Mg and Ca/Mg ratios, Cu and Zn, total protein, albumin, transferrin, glucose and lipid metabolism, growth factors, liver, kidney, and immune systems. These results should alert us to an increased risk of developing altered Vit-D, Ca, and P levels, conditions that we can prevent.

A limitation of this study is the small number of participants. Even though the study design proposed the nutritional status of children and adolescents with chronic diseases at nutritional risk in the pediatric service, including each pediatric specialty, to obtain a substantial sample of patients, in the end, the total number of patients was 95. It is the reason why we present only patients with malnutrition, syndromic diseases, encephalopathies, renal diseases, hyperlipidemia, IDDM, and eating disorders. The results of the remaining patients, 17 patients with CF, have been previously published [18]. Furthermore, an issue we could not address to complete the assessment of these nutrients was the assessment of dietary P intake. Nevertheless, notwithstanding the relatively limited sample, this work offers valuable and detailed information on the levels of Ca, P, Ca/P ratio, and Vit-D in this series of children and adolescents with chronic health conditions. The strengths of this study lie in the determination of the levels of Ca, P, Vit-D, the Ca/P ratio, and their relationship with anthropometric parameters, body composition, biochemical, and dietary indicators, in addition to the evaluation of the health and nutrition status in these patients.

The findings of this study have many practical implications in clinical practice and support the need to evaluate dietary, serum Ca, P, and Vit-D levels at least annually. It is important to perform blood and urine studies to confirm that the levels of these nutrients are adequate to make the adequate changes in their diet or to add supplements. Further studies, which take these variables into account, will need to be undertaken. A natural progression of this work is to implement multicenter trials to improve knowledge of Ca, P, and Vi-D to determine the necessary and appropriate amount of supplementation for effective prevention with personalized nutritional recommendations.

## 5. Conclusions

In this series of children and adolescents with chronic diseases, dietary calcium and vitamin D were deficient. Most patients had serum calcium levels in a regular range. There were no hypocalcemic and hyperphosphatemic patients. There was a simultaneous risk of vitamin D deficiency/insufficiency and hypophosphatemia. Our results support that there was an imbalance in Ca, P, and Vit-D metabolism in these patients and all nutrients were related to adiposity. Furthermore, we found a significant association between calcium, phosphorus, calcium/phosphorus ratio, and vitamin D levels with anthropometric parameters, body mass index, body composition, physical activity, diet, growth hormones, micronutrients and the immune, liver, and kidney systems.

## Figures and Tables

**Table 1:** Baseline characteristics of children with chronic disease by body mass index groups (*n* = 78) [6,16,17].

Characteristics	Total (*n* = 78)	Obesity (*n* = 24)	Undernutrition (*n* = 30)	Eutrophic (*n* = 24)	*p*-Value
	Mean ± SD	Mean ± SD	Mean ± SD	Mean ± SD	
Body fat percentage	21.7 ± 11.9	35.6 ± 5.0	11.4 ± 5.2	20.6 ± 8.8	<0.001 *
Fat mass index	4.9 ± 4.3	10.2 ± 3.2	1.6 ± 0.9	4.0 ± 2.3	<0.001 *
Fat-free mass index	14.6 ± 3.0	17.9 ± 2.9	12.0 ± 0.9	14.4 ± 0.9	<0.001 *
IGF-1 (ng/mL)	212 ± 136	264 ± 119	149 ± 115	241 ± 152	0.004 *
IGFBP3 (µg/mL)	2.8 ± 0.8	3.2 ± 0.8	2.4 ± 0.8	2.9 ± 0.8	0.004 *
Basal energy expenditure	954 ± 454	1283 ± 350	610 ± 324	1014 ± 199	0.000 *
Vitamin C (%DRI)	183 ± 212	256 ± 340	106 ± 77	202 ± 112	0.030 *
Vitamin D (%DRI)	107 ± 289	86 ± 177	82 ± 113	157 ± 474	0.589
Folic acid (%DRI)	167 ± 86	189 ± 83	133 ± 70	187 ± 96	0.024 *
Calcium intake (%DRI)	102 ± 37	94 ± 28	103 ± 34	110 ± 46	0.338
Vitamin D (ng/mL)	23.1 ± 13.6	17.7 ± 7.8	26.2 ± 16.3	24.9 ± 13.2	0.054
Serum calcium (mg/dL)	9.9 ± 0.4	9.8 ± 0.4	10 ± 0.4	9.8 ± 0.4	0.118
Serum phosphorus (mg/dL)	4.8 ± 0.6	4.8 ± 0.6	4.9 ± 0.6	4.6 ± 0.5	0.233
Serum calcium/phosphorus ratio	2.1 ± 0.2	2.1 ± 0.3	2.1 ± 0.2	2.1 ± 0.2	0.503
Deficient vitamin D intake (%)	58 (74.3)	19 (24.3)	21 (27)	18 (23.0)	
Deficient calcium intake (%)	27 (34.6)	11 (15.0)	7 (8.9)	9 (11.5)	
Deficiency serum vitamin D (%)	37 (47.4)	16 (20.5)	11 (14.1)	10 (12.9)	
Insufficiency serum vitamin D (%)	29 (37.2)	8 (10.2)	13 (16.7)	8 (10.2)	
Hypophosphatemia (%)	29 (37.2)	7 (8.9)	10 (12.9)	12 (15.4)	

Legend: IGF-1: insulin-like growth factor-1. IGFBP3: insulin-like growth factor-binding protein 3. DRI: Dietary Reference Intake. * *p* < 0.05: significant difference between groups by body mass index.

**Table 2:** Differences between participants with chronic diseases (*n* = 78).

Gender	Female (*n* = 43)	Male (*n* = 35)	*p*-Value
Serum vitamin D (ng/mL)	27.4 ± 16.6	19.7 ± 9.5	0.020
**Age group**	**Children (*n* = 42)**	**Adolescents (*n* = 36)**	
Calcium intake (%DRI)	119 ± 36	83 ± 27	<0.001
Body fat percentage	16.5 ± 10.9	27.7 ± 10.2	<0.001
Fat mass index	3.1 ± 2.9	7.1 ± 4.9	<0.001
Fat-free mass index	13.3 ± 7.8	16.1 ± 3.5	<0.001
Serum calcium (mg/dL)	10.0 ± 0.4	9.8 ± 0.4	0.015
Serum phosphorus (mg/dL)	5.0 ± 0.5	4.5 ± 0.5	<0.001
Serum calcium/phosphorus ratio	2.01 ± 0.17	2.21 ± 0.26	0.000
Insulin-like growth factor -1 (ng/mL)	143 ± 125	288 ± 105	0.000
Insulin-like growth factor-binding protein 3 (µg/mL)	2.4 ± 0.8	3.2 ± 0.6	0.000
**Serum vitamin D**	**Deficiency (*n* = 36)**	**Normal (*n* = 42)**	
Zinc (% Dietary Reference Intake)	80 ± 42	59 ± 27	0.010
Weight for age (kg)	45 ± 26	33 ± 25	0.041
Height for age (cm)	139 ± 27	124 ± 32	0.037
Fat mass (kg) anthropometry	13.8 ± 12.3	8.2 ± 9.7	0.029
Body fat percentage	25.6 ± 12.3	18.0 ± 10.6	0.002
Fat mass index	6.2 ± 4.7	3.8 ± 3.2	0.007
Waterlow I (%)	115.2 ± 38	98.7 ± 29	0.033
Serum vitamin D (mg/dL)	13.7 ± 3.8	31.4 ± 13.7	0.000
Serum magnesium (mg/dL)	2.03 ± 0.17	2.13 ± 0.19	0.026
**Calcium intake (% Dietary Reference Intake)**	**Deficient (*n* = 28)**	**Normal (*n* = 50)**	
Weight for age (kg)	51 ± 28	32 ± 22	0.001
Height for age (cm)	145 ± 27	125 ± 30	0.005
Hip circumference (cm)	86 ± 25	71 ± 22	0.009
Body mass index	22 ± 8	18 ± 6	0.037
Fat mass (kg) anthropometry	16.2 ± 12.9	8.2 ± 9.2	0.006
Fat-free mass (kg) anthropometry	35.3 ± 15.6	24.2 ± 14.2	0.002
Fat mass bioelectric impedancia analysis	17.1 ± 13.3	8.9 ± 8.6	0.008
Body fat percentage	25.6 ± 12.5	19.9 ± 11.2	0.023
Fat mass index	6.5 ± 4.6	4.3 ± 3.9	0.016
Fat-free mass index	15.6 ± 3.3	14.1 ± 2.8	0.027
Magnesium (% Dietary Reference Intake)	86 ± 29	115 ± 39	0.000
Serum phosphorus (mg/dL)	4.52 ± 0.52	4.93 ± 0.55	0.002
Serum calcium/phosphorus ratio	2.21 ± 0.27	2.04 ± 0.21	0.003
Copper (mg/dL)	106.2 ± 25.9	123.8 ± 29.6	0.011
Insulin-like growth factor-1 (ng/mL)	258 ± 127	189 ± 136	0.033
Leucocytes (cell/mm^3^)	6378 ± 2286	8024 ± 2024	0.002
Lymphocytes (cell/mm^3^)	2462 ± 904	3373 ± 1551	0.002
Platelets (cell/mm^3^)	257 ± 75	309 ± 92	0.014
Calcium/magnesium intake ratio	0.84 ± 0.25	1.18 ± 0.56	0.000
**Serum phosphorus**	**Deficiency (*n* = 28)**	**Normal (*n* = 50)**	
Weight for age (kg)	46 ± 28	34 ± 24	0.040
Height for age (cm)	142 ± 31	124 ± 30	0.018
Fat-free mass (kg) anthropometry	33.1 ± 16.3	24.5 ± 14.7	0.020
Zinc (% Dietary Reference Intake)	57 ± 24	77 ± 39	0.019
Serum calcium (mg/dL)	9.8 ± 0.3	10.0 ± 0.04	0.032
Serum calcium/phosphorus ratio	2.35 ± 0.20	1.96 ± 0.12	0.000
Creatinine (mg/dL)	0.58 ± 0.23	0.45 ± 0.15	0.006
Copper (mg/dL)	108.4 ± 26.1	123.7 ± 30.1	0.028
Leucocytes (cell/mm^3^)	6425 ± 1864	8030 ± 2271	0.002
Lymphocytes (cell/mm^3^)	2597 ± 871	3351 ± 1640	0.011
Platelets (cell/mm^3^)	256 ± 74	309 ± 88	0.006
**Erythrocyte sedimentation rate**	**Normal (*n* = 56)**	**High (*n* = 22)**	
Serum calcium (mg/dL)	9.9 ± 0.4	10.1 ± 0.4	0.045

**Table 3:** Significant odds ratios based on deficient serum and dietary vitamin D intake, deficient calcium intake, hypophosphatemia, stunted growth, and underweight throughout the series (*n* = 78).

	Fisher’s Exact Test	Odds Ratio	95% Confidence Interval		Cochran’s	Mantel–Haenszel
			Lower	Upper		
**Deficient vitamin D intake**						
Deficient lipid intake	0.037	6.786	0.835	55.166	0.043	0.091
Deficient kilocalories intake	0.037	4.500	0.944	21.441	0.044	0.087
Deficient carbohydrate intake	0.014	3.714	1.272	10.842	0.013	0.028
Deficient vitamin E intake	0.014	3.714	1.272	10.842	0.013	0.028
**Serum vitamin D deficiency**						
Excess body adiposity	0.031	2.727	1.054	7.058	0.036	0.065
Age-for-50° height < 5-year-old	0.020	1.436	1.048	1.967	0.022	0.042
**Deficient calcium intake**						
Deficit vitamin A intake	<0.001	6.071	2.133	17.279	<0.001	<0.001
Deficit kilocalories intake	0.003	4.875	1.675	14.190	0.003	0.006
Deficit magnesium intake	0.008	4.200	1.439	12.262	0.007	0.015
Higher weight for height	0.046	3.071	0.992	9.514	0.046	0.091
Adolescents	<0.001	2.910	1.802	4.700	<0.001	<0.001
Deficit carbohydrate intake	0.034	2.857	1.026	7.954	0.041	0.073
Deficit vitamin E intake	0.034	2.857	1.026	7.954	0.041	0.073
Females	0.034	1.530	1.038	2.254	0.040	0.072
Age-for-50° height ≥ 5-year-old	0.019	1.420	1.078	1.870	0.023	0.045
**Hypophosphatemia**						
Microcephalia	0.008	4.529	1.444	14.208	0.007	0.016
Deficit vitamin A intake	0.009	3.647	1.348	9.865	0.009	0.019
Deficit kilocalories intake	0.023	3.438	1.168	10.118	0.021	0.044
Deficit magnesium intake	0.023	3.438	1.168	10.118	0.021	0.044
Adolescents	0.014	1.815	1.133	2.908	0.015	0.029
Age-for-50° height ≥ 5-year-old	0.028	1.408	1.048	1.891	0.033	0.062
**Stunted growth**						
Wasting	<0.001	19.800	4.673	83.901	<0.001	<0.001
**Underweight**						
Stunting	<0.001	5.029	2.674	9.457	<0.001	<0.001

**Table 4:** Regression analysis between studied parameters with nutritional indicators in the whole series (*n* = 78).

Serum Vitamin D (ng/mL)	Calcium Intake (%DRI)	Serum Calcium (mg/dL)	Serum Phosphorus (mg/dL)	Serum Ca/P Ratio
***Linear***	***regression***	***analysis***		
Age (years) *R*^2^ = 0.085, *p* = 0.010	Age (years) *R*^2^ = 0.087, *p* = 0.009	Age (years) *R*^2^ = 0.135, *p* = 0.001	Age (years) *R*^2^ = 0.293, *p* = <0.001	Age (years) *R*^2^ = 0.215, *p* = <0.001
Hip circumference *R*^2^ = 0.098, *p* = 0.008	Hip circumference *R*^2^ = 0.085, *p* = 0.012	Head circumference *R*^2^ = 0.128, *p* = 0.002	Height for age *R*^2^ = 0.187, *p* = <0.001	MAMC *R*^2^ = 0.178, *p* = 0.004
Body fat percentage *R*^2^ = 0.079, *p* = 0.013	Body fat percentage *R*^2^ = 0. 067, *p* = 0.023	Fat-free mass index *R*^2^ = 0.083, *p* = 0.012	Body mass index *R*^2^ = 0.078, *p* = 0.014	Disease duration *R*^2^ = 0.054, *p* = 0.043
FFM kg by BIA *R*^2^ = 0.085, *p* = 0.047	FM by BIA *R*^2^ = 0.141, *p* = 0.008	FFM kg by A *R*^2^ = 0.121, *p* = 0.004	TTSPA *R*^2^ = 0.103, *p* = 0.012	TTSPA *R*^2^ = 0.096, *p* = 0.015
Energy expenditure *R*^2^ = 0.090, *p* = 0.041		Energy expenditure *R*^2^ = 0.163 *p* = 0.006	Energy expenditure *R*^2^ = 0.122, *p* = 0.019	
Vitamin B2 (%DRI) *R*^2^ = 0.071, *p* = 0.020		Fiber (%DRI) *R*^2^ = 0.071, *p* = 0.021		Serum phosphorus *R*^2^ = 0.851, *p* = <0.001
Triglycerides *R*^2^ = 0.116, *p* = 0.004	IGF-1 *R*^2^ = 0.052, *p* = 0.050		IGF-1 *R*^2^ = 0.083, *p* = 0.012	AST *R*^2^ = 0.119, *p* = 0.004
		Lymphocytes *R*^2^ = 0.211, *p* = <0.001		Lymphocytes *R*^2^ = 0.100, *p* = 0.005
***Multilinear***	***regression***	***analysis***		
			FFM kg by A and MAMC *R*^2^ = 0.261, *p* = 0.001	Weight for age and waist circumference *R*^2^ = 0.163, *p* = 0.002
	Ca/Mg intake ratio and magnesium (%DRI) *R*^2^ = 0.812, *p* = <0.001		Protein, fiber, and magnesium (%DRI) *R*^2^ = 0.224, *p* = 0.004	Protein and zinc (%DRI) *R*^2^ = 0.207, *p* = <0.001
Vitamin C and serum magnesium *R*^2^ = 0.200, *p* = <0.001	Serum vitamin B 12 and phosphorus *R*^2^ = 0.243, *p* = <0.001	Serum phosphorus and Mg/Ca ratio *R*^2^ = 0.379, *p* = <0.001	Serum Ca/P ratio and serum calcium *R*^2^ = 0.982, *p* = <0.001	
		Creatinine, albumin, alkaline phosphatase *R*^2^ = 0.330, *p* = <0.001	AST and creatinine *R*^2^ = 0.231, *p* = <0.001	
Leucocytes and IgG3 *R*^2^ = 0.201, *p* = 0.002	Lymphocytes and CD16 + 56 T-lymphocytes *R*^2^ = 0.204, *p* = <0.001		Lymphocytes and platelets *R*^2^ = 0.266, *p* = <0.001	

Legend: Ca: calcium. P: phosphorus. MAMC: mid-arm muscle circumference. FFM: fat-free mass. FM: fat mass. BIA: bioelectrical impedance analysis. A: anthropometry. TTSPA: total time spent on physical activity. DRI: Dietary Reference Intake. IGF-1: insulin-like growth factor-1. AST: aspartate amino transferase. Mg: magnesium. Ig: immunoglobulin.

## Data Availability

Data are contained within the article and Supplementary Materials. Data supporting the reported results can also be found in references [6,16,17].

## Supplementary Materials

The following supporting information can be downloaded at: https://www.mdpi.com/article/10.3390/nu16091349/s1, Table S1: Significant correlations between serum calcium intake, serum calcium, phosphorus, and calcium/phosphorus ratio with nutritional indicators in the whole series (*n* = 78); Table S2: Correlations between serum vitamin D, serum calcium, dietary calcium intake, serum phosphorus, and serum calcium/phosphorus ratio with nutritional indicators by body mass index groups (*n* = 78), ** *p* < 0.01 (2 tailed). Table S3: Regression analysis between serum and dietary calcium and vitamin D intake, serum phosphorus, serum calcium/phosphorus ratio, and nutritional parameters by body mass index groups (*n* = 78).

## Abbreviations

PA	Physical activity
BMI	Body mass index
BF%	Body fat percentage
FFM	Fat-free mass
FM	Fat mass
BIA	Bioelectrical impedance analysis
%DRI	% Dietary Reference Intake
FUNIBER	Fundación Universitaria Iberoamericana
WHO	World Health Organization
NHANES	National Health and Nutrition Examination Survey
EsNuPI	Estudio Nutricional en Población Infantil Española
ANIVA	Antropometría y Nutrición Infantil de Valencia
ANIBES	Anthropometry, Intake, and Energy Balance in Spain
IDEFICS	Identification and prevention of dietary- and lifestyle-induced health effects in children and infants
