# Supplementary material for: Calcium, Phosphate, and Vitamin D in Children and Adolescents with Chronic Diseases: A Cross-Sectional Study
Source: Nutrients. 2024 Apr 29;16(9):1349. doi: 10.3390/nu16091349 (PMC11085162; doi:10.3390/nu16091349)
Supplement: Supplementary file 1 [file nutrients-16-01349-s001.zip › Supplementary Materials_nutrients-2952266.pdf]

nutrients

MDPI

Supplementary Material

Calcium, Phosphate, and Vitamin D in Children and Adolescents with Chronic Diseases: A Cross-Sectional Study

Marlene Fabiola Escobedo-Monge [1,*], Pilar Bahillo-Curieses [2], Joaquín Parodi-Román [3], María Antonieta Escobedo-Monge [4], Pedro Alonso López[5], and José Manuel Marugán-Miguelsanz [1,5]

1 Department of Pediatrics of the Faculty of Medicine, University of Valladolid, Avenida Ramón y Cajal, 7, 47005 Valladolid, Spain.
2 Section of Pediatric Endocrinology, University Clinical Hospital of Valladolid, Avenida Ramón y Cajal, 7, 47005 Valladolid, Spain; pilarbahilloc@yahoo.es
3 Science Faculty, University of Cadiz, Paseo de Carlos III, 28, 11003 Cádiz; joaquin_parodi@yahoo.es
4 Department of Chemistry, Science Faculty, University of Burgos, Plaza Misael Bañuelos s/n, 09001 Burgos, Spain; antoitalia777@gmail.com
5 Section of Gastroenterology and Pediatric Nutrition, University Clinical Hospital of Valladolid, Avenida Ramón y Cajal, 7, 47005 Valladolid, Spain; jmmarugan@telefonica.net; palonsolo@saludcastillayleon.es
* Correspondence: amescobedo@msn.com; Tel.: +34-639-590-467

Table S1. Significant correlations between serum calcium intake, serum calcium, phosphorus, and calcium/phosphorus ratio with nutritional indicators in the whole series (n = 78).

Correlations	Serum Vitamin D		Calcium intake		Serum calcium		Serum phosphorus		Serum Ca/P ratio	
	r	*p-value*	*r*	*p-value*	*r*	*p-value*	*r*	*p-value*	*r*	*p-value*
Age (years)	-0.292**	0.010	-0.295**	0.009	-0.368**	0.001	-0.541**	<0.001	0.464**	<0.001
Weight-for-age (kg)	-0.286*	0.012	-0.268*	0.018	-0.379**	<0.001	-0.414**	<0.001	0.314**	0.006
Weight-for-age Z-score	-0.290*	0.011	-0.264*	0.020	-0.279*	0.015				
Height-for-age (cm)	-0.303**	0.007	-0.272*	0.017	-0.405**	<0.001	-0.439**	<0.001	0.321**	0.005
Age-for-50ºHeight	-0.273*	0.016			-0.368**	0.001	-0.404**	<0.001	0.298**	0.009
Weight-for-Height Z-score	-0.296**	0.009			-0.259*	0.024				
Cephalic circumference (cm)	-0.299**	0.008			-0.453**	<0.001	-0.381**	<0.001	0.236*	0.040
Wrist circumference (cm)	-0.303**	0.009			-0.290*	0.013				
Waist circumference (cm)	-0.300**	0.010			-0.290*	0.013	-0.318**	0.006	0.250*	0.034
Waist circumference Z-s	-0.279*	0.017			-0.280*	0.017				
Hip circumference (cm)	-0.312**	0.007	-0.291*	0.012	-0.315**	0.007	-0.396**	<0.001	0.317**	0.006
Hip circumference Z-score	-0.283*	0.015			-0.279*	0.017				
MUAC (cm)	-0.258*	0.023			-0.334**	0.003	-0.271*	0.018		
MUAC Z-score	-0.256*	0.025			-0.263*	0.022				
Bicipital skinfold Z-score	-0.267*	0.019								
Triceps skinfold Z-score			-0.239*	0.036			-0.307**	0.007	0.280*	0.014
Subscapular skinfold Z-score	-0.264*	0.020								
Sum of skinfolds (mm)	-0.259*	0.023								
Body mass index	-0.257*	0.024			-0.277*	0.015	-0.280*	0.014		
Body mass index Z-score	-0.264*	0.020								
Nutritional index	-0.260*	0.022			-0.255*	0.026				
Waterlow I (%)	-0.247*	0.030			-0.237*	0.039				
Waterlow II (%)	-0.229*	0.045			-0.267*	0.020				
Body fat percentage	-0.281*	0.013	-0.259*	0.023	-0.243*	0.035	-0.268*	0.019		
Fat mass index	-0.272*	0.017	-0.228*	0.046	-0.254*	0.027	-0.278*	0.015		
Fat free mass index	-0.227*	0.047			-0.288*	0.012	-0.252*	0.028		
Fat mass by A	-0.281*	0.013	-0.259*	0.023	-0.242*	0.035	-0.268*	0.019		
Fat mass kg by A	-0.267*	0.019	-0.265*	0.020	-0.313**	0.006	-0.360**	0.001	0.288*	0.012
Fat free mass by A	0.281*	0.013	0.259*	0.023	0.242*	0.035	0.268*	0.019		
Fat free mass kg by A	-0.282*	0.013	-0.254*	0.026	-0.404**	<0.001	-0.428**	<0.001	0.313**	0.006
Fat mass by BIA	-0.279*	0.019	-0.313**	0.008	-0.301*	0.012	-0.351*	0.003	0.304*	0.011
Fat free mass by BIA	-0.300*	0.012					-0.326**	0.006		

Far free mass kg by BIA	-0.289*	0.044					-0.380**	0.008	0.303*	0.037
Arm area	-0.276*	0.016			-0.304**	0.008	-0.335**	0.003	0.265*	0.021
Arm muscle area	-0.243*	0.033			-0.360**	0.001	-0.305**	0.007		
Arm fat area	-0.272*	0.017			-0.234*	0.042	-0.333**	0.003	0.300**	0.008
MUAC							-0.290*	0.011	0.355**	0.002
Muscle area/Fat area index	-0.267*	0.019	-0.228*	0.046			-0.269*	0.019	0.275*	0.016
Muscle fat coefficient			-0.234*	0.041			-0240*	0.037	0.242*	0.035
Energy expenditure	-0.299*	0.041			-0.404**	0.006	-0.349*	0.019		
TTSPA							-0.321*	0.012	0.310*	0.015
Kilocalories (%DRI)			0.379**	<0.001	-0.332**	0.004			-0.234*	0.044
Protein (%DRI)			0.349**	0.002			0.330**	0.004	-0.379**	<0.001
Fiber (%DRI)					-0.277*	0.016	-0.271*	0.019		
Cholesterol (%DRI)							-0.239*	0.039		
Vitamin B2 (%DRI)	0.266*	0.020			0.250*	0.030				
Vitamin B6 (%DRI)	0.255*	0.026								
Calcium (%DRI)							0.241*	0.038		
Magnesium (%DRI)			0.370**	<0.001			0.266*	0.021	-0.244*	0.035
Ca/Mg intake ratio			0.538**	<0.001						
Zinc (%DRI)									-0.282*	0.014
Iron (%DRI)			0.347**	0.002						
Beta carotene (μg/L)							0.260*	0.025		
Folic acid (mg/mL)	0.243*	0.037								
Serum vitamin B12 (pg/dL)			0.438**	<0.001						
Serum vitamin C (mg/L)	0.332**	0.005			0.238*	0.049				
Serum Ca/P ratio							-0.923**	<0.001		
Serum calcium (mg/dL)							0.492**	<0.001		
Serum phosphorus (mg/dL)			0.241*	0.038	0.492**	<0.001			-0.923**	<0.001
Serum copper (μg/dL)							0.285*	0.013	-0.255*	0.026
Serum copper/zinc ratio							0.226*	0.050		
Serum magnesium (mg/dL)	0.274*	0.019								
Serum Ca/Mg ratio					0.319**	0.006				
Serum Mg/Ca ratio					-0.344*	0.033				
Transferrin (mg/dL)					0.230*	0.049				
Ferritin (ng/mL)					-0.306**	0.008				
Glucose (mg/dL)					-0.304**	0.008	-0.303**	0.008	0.256*	0.026
Creatinine (mg/dL)					-0.327**	0.004	-0.428**	<0.001	0.343**	0.002
Total protein					0.345**	0.002				
Albumin (mg/dL)					0.294*	0.014				
Triglycerides (mg/dL)	0.332**	0.003								
Total bilirubin (mg/dL)							-0.257*	0.034		
AST (U/L)	0.305*	0.011			0.310*	0.010	0.424**	<0.001	0.346**	0.004
ALT (U/L)					-0.275*	0.023				
MCV (μg/m^3)					-0.256*	0.026				
Leucocytes (cell/mm^3)	0.284*	0.012	0.301**	0.008	0.379**	<0.001	0.407**	<0.001	-0.297**	0.009
Lymphocytes (cell/mm^3)			0.353**	0.002	0.459**	<0.001	0.459**	<0.001	-0.316**	0.005
Platelets (cell/mm^3)					0.250*	0.029	0.363**	0.001	-0.278*	0.015
IgG3 (mg/dL)	0.303*	0.021								
IgA (mg/dL)							-0.276*	0.027		
CD16+56 T-lymphocytes			-0.314*	0.011						
IGF-1 (ng/mL)			-0.228*	0.050			-0.287*	0.012		
Disease duration									0.233*	0.043

Legend: Ca: calcium. P: phosphorus. A: anthropometry. BIA: bioelectric impedance analysis. MUAC: mid upper-arm circumference. MAMC: mid arm muscle circumference. %DRI: dietary reference intake. Mg: magnesium. AST: aspartate aminotransferase. ALT: alanine aminotransferase. MCV: Mean corpuscular volume. IGF-1: insulin-like growth factor-1. * $p < 0.05$** $p < 0.01$ (2 tailed).

Table S2. Correlations between serum vitamin D, serum calcium, dietary calcium intake, serum phosphorus and serum calcium/phosphorus ratio with nutritional indicators by body mass index groups (n = 78), ** $p < 0.01$ (2 tailed).

	**Serum Vitamin D**		**Serum Calcium**	
**Obese**	*r*	*p-value*	*r*	*p-value*
Waist/hip ratio	-0.543**	0.009		
Ferritin (ng/mL)	-0.552**	0.006		
CD8 T-Lymphocytes			0.610**	0.009
**Undernutrition**				
Serum phosphate (mg/dL)			0.618**	<0.001
Transferrin (mg/dL)			0.483**	0.008
Medium corpuscular volume (µg/m3)			-0.487**	0.006
CD16+56 T Lymphocytes	-0.515**	0.007		
**Eutrophic**				
Height-for-age Z-score	-0.585**	0.003		
Waterlow II (%)	-0.606**	0.002		
Insulin-like growth factor-binding protein-3 (mg/L)	-0.592**	0.003		
Complement C3			0.596**	0.006

	**Calcium**	**Intake**	**Serum**	**P**	**Serum**	**Ca/P r**
**Obese**	*r*	*p-value*	*r*	*p-value*	*r*	*p-value*
Age (years)			-0.699**	<0.001	0.636**	0.001
Weight-for-age (kg)			-0.606**	0.002		
Height-for-age (cm)			-0.595**	0.003		
Head circumference (cm)			-0.563**	0.005		
Waist circumference (cm)			-0.583**	0.006	0.566**	0.008
Hip circumference (cm)			-0.667**	<0.001	0.620**	0.002
Suprailiac skinfold (mm)					0.528**	0.010
Triceps skinfold (mm)					0.592**	0.003
Mid upper-arm circumference (cm)			-0.557**	0.006		
Fat mass kg Anthropometry			-0.632**	0.001	0.576**	0.004
Fat free mass kg Anthropometry			-0.641**	<0.001		
Fat mass Bioelectric Impedance Analysis	-0.587**	0.003	-0.701**	<0.001	0.659**	<0.001
Fat mass kg Bioelectric Impedance Analysis	-0.705**	<0.001	-0.584**	0.007	0.670**	0.001
Fat free mass Bioelectric Impedance Analysis			-0.559**	0.006		
Fat free mass kg Bioelectric Impedance Analysis			-0.594**	0.006		
Arm area			-0.544**	0.007		
Fat area arm			-0.572**	0.004	0.604**	0.002
Mid arm muscle circumference			-0.579**	0.004		
Body mass index			-0.539**	0.008		
Fat mass index			-0.485*	0.019	0.450*	0.031
Fat free mass index			-0.498*	0.016		
Vitamin B12 (%Dietary Reference Intake)	0.608**	0.002				
Magnesium (%Dietary Reference Intake)	0.585**	0.003	0.633**	0.001	-0.596**	0.003
Iron (%Dietary Reference Intake)	0.698**	<0.001			-0.548**	0.007
Zinc (%Dietary Reference Intake)			0.565**	0.005	-0.646**	<0.001
Beta-carotene (µg/L)			0.527**	0.010		
Copper (µg/dL)			0.729**	<0.001	-0.612**	0.002
Serum Calcium/Phosphorus ratio			-0.913**	<0.001		
Serum Copper/Zinc ratio			0.590**	0.003	-0.576**	0.004
Serum Zinc/Copper ratio			-0.594**	0.003	0.578**	0.004
Prealbumin (mg/dL)			-0.555**	0.009		
Creatinine (mg/dL)			-0.706**	<0.001	0.642**	<0.001
Leucocytes (cell/mm^3)	0.573**	0.003				
Lymphocytes (cell/mm^3)	0.560**	0.004				

**Undernutrition**						
Age (years)	-0.369*	0.045	-0.476**	0.008	0.401*	0.028
Arm fat area			-0.495**	0.005	0.500**	0.005
Body mass index Z-score	0.535**	0.003				
Body fat percentage			-0.429*	0.018	0.434*	0.016
Fat mass index			-0.395*	0.031	0.381*	0.038
Kilocalories (%Dietary Reference Intake)	0.527**	0.003				
Protein (%Dietary Reference Intake)	0.535**	0.003			-0.551**	0.002
Carbohydrates (%Dietary Reference Intake)	0.537**	0.003				
Vitamin A (%Dietary Reference Intake)	0.484**	0.008	0.522**	0.004	0.521**	0.004
Magnesium (%Dietary Reference Intake)	0.478**	0.009				
Iron (%Dietary Reference Intake)	0.596**	<0.001				
Iodine (%Dietary Reference Intake)	0.544**	0.002				
Serum phosphate (mg/dL)	0.500**	0.006				
Serum Calcium/Phosphorus ratio	-0.477**	0.009	-0.909**	<0.001		
Leucocytes (cell/mm^3)			0.475**	0.008	-0.481**	0.007
Lymphocytes (cell/mm^3)			0.523**	0.003	-0.484**	0.007
**Eutrophic**						
Age (years)			-0.432*	0.026	0.423*	0.044
Fat mass kg BIA					0.581**	0.009
Fat mass index			-0.456*	0.029	0.458*	0.028
Fat free mass index			-0.575**	0.004	0.555**	0.006
Carbohydrates (%Dietary Reference Intake)			0.562**	0.005		
Serum Calcium/Phosphorus ratio			-0.945**	<0.001		
Albumin (mg/dL)	-0.564**	0.005				
Aspartate aminotransferase (U/L)			0.567**	0.006	-0.538**	0.010
Monocyte (%)			0.556**	0.006		

Table S3. Regression analysis between serum and dietary calcium and vitamin D intake, serum phosphorus, serum calcium/phosphorus ratio and nutritional parameters by body mass index groups ($n = 78$).

**Obesity (n = 24)**		**Undernutrition (n= 30)**		**Eutrophic (n = 24)**	
**Serum vitamin D**	**Vitamin D intake**	**Serum vitamin D**	**Vitamin D intake**	**Serum vitamin D**	**Vitamin D intake**
Linear	*Regression*	*Analysis*			
Waist/hip ratio<br>$R^2 = 0.317$, $p = 0.008$<br>Serum vitamin C<br>$R^2 = 0.247$, $p = 0.016$		FFM kg BIA<br>$R^2 = 0.600$, $p = 0.024$<br>Serum vitamin C<br>$R^2 = 0.183$, $p = 0.037$<br>Triglycerides<br>$R^2 = 0.345$, $p = 0.001$		Height-for-age Z-s<br>$R^2 = 0.273$, $p = 0.011$<br>Waterlow II<br>$R^2 = 0.316$, $p = 0.005$<br>Serum magnesium<br>$R^2 = 0.271$, $p = 0.022$<br>IGFBP3<br>$R^2 = 0.247$, $p = 0.016$	
Multilinear	*Regression*	*Analysis*			
		IgG3 and CD16+56 T-lymphocytes<br>$R^2 = 0.629$, $p = < 0.001$			
**Serum calcium**	**Calcium intake**	**Serum calcium**	**Calcium intake**	**Serum calcium**	**Calcium intake**
Linear	*Regression*	*Analysis*			
MAMC<br>$R^2 = 0.216$, $p = 0.026$<br>Serum Mg/Ca ratio<br>$R^2 = 0.317$, $p = 0.005$	FM by BIA<br>$R^2 = 0.375$, $p = 0.003$<br>Serum vitamin B12<br>$R^2 = 0.271$, $p = 0.011$	Transferrin<br>$R^2 = 0.218$, $p = 0.016$	FA/MA index<br>$R^2 = 0.254$, $p = 0.017$<br>Lymphocytes<br>$R^2 = 0.229$, $p = 0.016$	Head circumference<br>$R^2 = 0.182$, $p = 0.042$	Hip circumference<br>$R^2 = 0.173$, $p = 0.043$<br>Albumin<br>$R^2 = 0.219$, $p = 0.024$<br>Serum vitamin B12<br>$R^2 = 0.213$, $p = 0.030$<br>Complement C3<br>$R^2 = 0.199$, $p = 0.049$
Multilinear	*Regression*	*Analysis*			
CD8 T-Lymphocytes and lymphocytes<br>$R^2 = 0.664$, $p = 0.001$	Mg, and Ca/Mg ratio (%DRI)<br>$R^2 = 0.795$, $p = < 0.001$	Serum phosphorus and Mg/Ca ratio<br>$R^2 = 0.590$, $p = < 0.001$<br>CD8 T-Lymphocytes and MCV<br>$R^2 = 0.480$, $p = 0.001$	BMI Z-score and FMI<br>$R^2 = 0.288$, $p = 0.012$<br>Iron, Mg and Ca/Mg ratio (%DRI)<br>$R^2 = 0.835$, $p = < 0.001$<br>Serum phosphorus and vitamin B12<br>$R^2 = 0.324$, $p = 0.006$	Glucose and creatinine<br>$R^2 = 0.440$, $p = 0.005$	Mg, and Ca/Mg ratio (%DRI)<br>$R^2 = 0.869$, $p = < 0.001$<br>CD16+56 T-lymphocytes and leucocytes<br>$R^2 = 0.473$, $p = 0.002$
**Serum phosphorus**	**Serum Ca/P ratio**	**Serum phosphorus**	**Serum Ca/P ratio**	**Serum phosphorus**	**Serum Ca/P ratio**
Linear	*Regression*	*Analysis*			
Age (years)<br>$R^2 = 0.699$, $p = < 0.001$<br>Hip circumference<br>$R^2 = 0.422$, $p = 0.001$<br>Body mass index<br>$R^2 = 0.312$, $p = 0.006$<br>Energy expenditure<br>$R^2 = 0.255$, $p = 0.023$<br>IGF-1<br>$R^2 = 0.214$, $p = 0.026$<br>Creatinine<br>$R^2 = 0.301$, $p = 0.028$	Age (years)<br>$R^2 = 0.636$, $p = 0.001$<br>Hip circumference<br>$R^2 = 0.304$, $p = 0.010$<br>Fat mass index<br>$R^2 = 0.202$, $p = 0.031$<br>MAMC<br>$R^2 = 0.396$, $p = 0.003$<br>Serum phosphorus<br>$R^2 = 0.853$, $p = < 0.001$<br>Protein (%DRI)<br>$R^2 = 0.272$, $p = 0.011$	Age (years)<br>$R^2 = 0.401$, $p = 0.028$<br>Head circumference<br>$R^2 = 0.171$, $p = 0.029$<br>Body fat percentage<br>$R^2 = 0.184$, $p = 0.018$<br>Arm area<br>$R^2 = 0.523$, $p = 0.043$<br>Vitamin A (%DRI)<br>$R^2 = 0.242$, $p = 0.007$<br>AST<br>$R^2 = 0.159$, $p = 0.044$<br>Lymphocytes<br>$R^2 = 0.290$, $p = 0.005$	Age (years)<br>$R^2 = 0.699$, $p = < 0.001$<br>Body fat percentage<br>$R^2 = 0.189$, $p = 0.016$<br>TTSPA<br>$R^2 = 0.204$, $p = 0.040$<br>Leucocytes<br>$R^2 = 0.600$, $p = 0.024$<br>Serum phosphorus<br>$R^2 = 0.200$, $p = 0.008$<br>Protein (%DRI)<br>$R^2 = 0.215$, $p = 0.011$<br>Creatinine<br>$R^2 = 0.170$, $p = 0.029$	Age (years)<br>$R^2 = 0.462$, $p = 0.026$<br>Carbohydrates (%DRI)<br>$R^2 = 0.354$, $p = 0.003$<br>Glucose<br>$R^2 = 0.358$, $p = 0.004$<br>Monocytes<br>$R^2 = 0.280$, $p = 0.016$	Age (years)<br>$R^2 = 0.423$, $p = 0.044$<br>Triceps Z-score<br>$R^2 = 0.331$, $p = 0.004$<br>Fat free mass index<br>$R^2 = 0.308$, $p = 0.006$<br>Arm fat area<br>$R^2 = 0.414$, $p = 0.004$<br>Serum phosphorus<br>$R^2 = 0.890$, $p = < 0.001$<br>Kilocalories (%DRI)<br>$R^2 = 0.245$, $p = 0.016$<br>Glucose<br>$R^2 = 0.296$, $p = 0.009$
Multilinear	*Regression*	*Analysis*			
FFM by A, MAMC<br>$R^2 = 0.438$, p = 0.007<br>Mg and fiber (%DRI)<br>$R^2 = 0.470$, $p = 0.002$<br>Serum Ca/P ratio and calcium<br>$R^2 = 0.985$, $p = <0.001$		Serum Ca/P ratio and calcium<br>$R^2 = 0.978$, $p = < 0.001$		WA and HA<br>$R^2 = 0.417$, $p = 0.005$<br>BMI and BF%<br>$R^2 = 0.547$, $p = < 0.001$<br>AFA and FFM by A<br>$R^2 = 0.500$, $p = 0.006$<br>Serum Ca/P ratio and calcium<br>$R^2 = 0.988$, $p = < 0.001$<br>MCV and monocytes<br>$R^2 = 0.467$, $p = 0.016$	

Legend: FFM: fat free mass. FM: fat mass. BIA: bioelectrical impedance analysis. Z-s: Z-score. IGFBP3: insulin-like growth factor-binding protein 3. Ig: immunoglobulin. MAMC: mid arm muscle circumference. FA: fat area. MA: muscle area. Mg: magnesium. Ca: calcium. P: phosphorus. BMI: Body mass index. FMI: fat mass index. DRI: Dietary Reference Intake. MCV: Mean corpuscular volume. TTSPA: Total time spent on physical activity. IGF-1: insulin-like growth factor-1. AST: Aspartate aminotransferase. WA: weight-for-age. HA: height-for-age. BF%: body fat percentage. AFA: arm fat area.
